# Mitochondrial stress activates ELT-2–dependent lysosomal proteostasis to extend lifespan in *C. elegans*

**DOI:** 10.1126/sciadv.aeh0657

**Published:** 2026-08-12

**Authors:** Rendan Yang, Yu Sun, Wenzheng Wang, Yuxuan Lei, Lin Chen, Xiaoxu Li, Raya B. Liu, Ang Zhou, Arwen W. Gao, Liangshan Mu, Johan Auwerx, Terytty Yang Li

**Affiliations:** ^1^ Shanghai Key Laboratory of Metabolic Remodeling and Health, Institute of Metabolism and Integrative Biology, Reproductive Medicine Center, Zhongshan Hospital, Obstetrics & Gynecology Hospital of Fudan University, Shanghai Key Lab of Reproduction and Development, Shanghai Key Lab of Female Reproductive Endocrine Related Diseases, Fudan University, Shanghai, China.; ^2^ Laboratory of Integrative Systems Physiology, Interfaculty Institute of Bioengineering, École Polytechnique Fédérale de Lausanne, Lausanne, Switzerland.; ^3^ Reproductive Medicine Center, Zhongshan Hospital, Fudan University, Shanghai, China.

## Abstract

Mild mitochondrial stress could extend lifespan across species, yet the underlying mechanism remains unclear. Here, we show that inhibition of mitochondrial respiration induces a sustained transcriptional program that enhances lysosomal proteolysis during aging in *Caenorhabditis elegans*. Mechanistically, this response is primarily regulated by the intestinal GATA transcription factor ELT-2, which retains high expression and directly binds to GATA motifs in the promoters of lysosomal protease genes to promote their transcriptional activation. Moreover, we identified R249 within the conserved zinc-finger DNA binding domain of ELT-2 as a key residue required for its transcriptional activity. Notably, this mitochondrion–ELT-2–lysosome axis operates largely independently of the mitochondrial unfolded protein response (UPR^mt^) to counteract aging. Furthermore, increased lysosomal activity, as well as the lysosomal proteases CPR-5 and CPR-8, is essential for mitochondrial stress–induced clearance of toxic polyglutamine (polyQ) aggregates and lifespan extension. Together, our findings reveal a previously unrecognized ELT-2–dependent lysosomal proteostasis pathway that acts downstream of mitochondrial stress to maintain protein homeostasis and promote longevity.

## INTRODUCTION

Mitochondria are multifaceted organelles that not only contribute to the harvesting of energy, but also serve as key signaling hubs connecting numerous metabolic processes to essential cellular and organismal functions (*1*–*3*). In general, mitochondrial dysfunction is detrimental and contributes to the pathogenesis of diverse age-related diseases, as well as severe early-onset inherited mitochondrial disorders (*1*, *4*–*6*). Intriguingly, mild mitochondrial stress, in some cases, could trigger a host of cellular changes that surpass the initial stress and lead to overall improved organismal fitness, a phenomenon termed mitohormesis (*7*). For instance, mitochondrial respiration inhibition by RNA interference (RNAi) of electron transport chain (ETC) components (*8*, *9*), mitochondrial translation components (*10*), TCA cycle enzymes (*11*), and mitochondrial protein transporters (*12*), extends lifespan by 32 to 87% in *Caenorhabditis elegans*. This phenomenon is furthermore highly conserved across eukaryotic species: in yeast, flies, and mice, mitochondrial perturbation could delay the aging process (*13*–*16*). Nevertheless, after more than two decades of investigation, the determining factor(s) that contribute to this process remain elusive or even controversial.

It was shown, and widely propagated, that the UPR^mt^ (*17*–*19*), which senses mitochondrial protein-folding stress, played a causal role in ETC inhibition–induced longevity (*10*, *20*, *21*). However, this model has been challenged recently, as complete deletion of *atfs-1*, which encodes a master transcription factor (TF) of the UPR^mt^ (*17*, *22*), strongly disrupted the UPR^mt^ but barely prevented the lifespan extension caused by RNAi-mediated silencing of ETC gene *cytochrome c oxidase* (*cco-1*) or *iron-sulphur protein* (*isp-1*)*(qm150)* mutation (*23*–*26*). In addition, constitutively active alleles of *atfs-1* activate the UPR^mt^ but fail to extend lifespan (*23*, *27*). These findings suggest that the UPR^mt^, at least the ATFS-1–mediated part, is neither necessary nor sufficient for lifespan extension in certain contexts in *C. elegans*.

Beyond the UPR^mt^, several additional signaling pathways and factors, including the reactive oxygen species (ROS)–induced hypoxia-inducible factor (HIF-1) pathway (*28*), the apoptotic pathway (*29*), homeobox protein CEH-23 (*30*), p53 ortholog CEP-1 (*31*), mitogen-activated protein kinase PMK-3 (*32*), and AMP-activated protein kinase (AMPK) (*33*), have been implicated in partially mediating lifespan extension downstream of mitochondrial perturbations. However, none of these factors was able to account for the full lifespan extension following ETC suppression such as that induced by *cco-1* RNAi, or the *isp-1(qm150)* mutation. Thus, a determining mechanism, if it exists, for the longevity phenotype in response to mitochondrial inhibition or stress remains to be revealed.

Lysosomes are central hubs for cellular clearance and metabolic signaling (*34*, *35*). Beyond their canonical role in degrading macromolecules via acidic hydrolases, lysosomes integrate stress signals and orchestrate adaptive transcriptional programs that maintain proteostasis (*36*). During aging, lysosomal function declines, manifested as reduced acidification, impaired autophagic flux, and diminished cathepsin activity, leading to accumulation of protein aggregates and damaged organelles, which contribute to age-related diseases (*35*). Conversely, genetic or pharmacological interventions that enhance lysosomal activity, such as Transcription factor EB (TFEB) overexpression or cathepsin activation (*37*, *38*), extend healthspan in multiple model organisms. In *C. elegans*, preservation of lysosomal proteolysis is associated with longevity paradigms, including dietary restriction and reduced insulin/insulin-like growth factor 1 signaling (*39*). However, whether and how lysosomal adaptation contributes specifically to the beneficial effects of mild mitochondrial stress has remained largely unexplored.

Here, by comprehensively analyzing the age-associated differentially expressed genes (DEGs) and pathways in response to mitochondrial respiration inhibition in *C. elegans*, we reveal that a lysosomal adaptive transcriptional program is activated under mitochondrial stress. Mechanistically, through an RNAi-based screen for possible regulatory factors, we identified the GATA TF ELT-2 as the master regulator of this response. We found that this mitochondrion–ELT-2–lysosome axis preserves lysosomal acidification and degradative capacity upon aging, reducing polyglutamine (polyQ) aggregates in *C. elegans* models of Huntington’s disease (HD). Intriguingly, suppression of lysosomal acidification, ELT-2 or the lysosomal proteases CPR-5/CPR-8, all potently abolishes mitochondrial inhibition-induced longevity. Together, these results uncover a central ELT-2–mediated mitochondrion-nucleus-lysosome communication mechanism that sustains proteostasis and extends organismal lifespan downstream of mitochondrial stress, and is hence a key effector arm of mitohormesis.

## RESULTS

### Mitochondrial stress remodels the expression of lysosomal genes upon aging

**Fig. 1.**
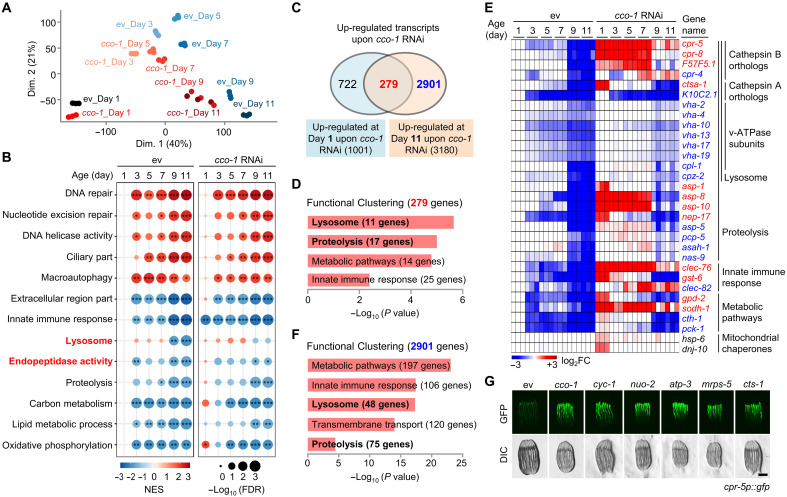
Mitochondrial respiration inhibition activates and sustains the expression of lysosome/proteolysis-related genes upon aging in *C. elegans*. (**A**) Principal components analysis (PCA) of the RNA-seq profiles of worms treated with control (empty vector, ev) or *cco-1* RNAi and harvested at days 1, 3, 5, 7, 9, and 11 of adulthood (*n* = 3 biological replicates for each condition). (**B**) Time-dependent tracking of the processes that are differentially regulated upon aging based on gene set enrichment analysis (GSEA). NES, normalized enrichment score. FDR, false discovery rate. (**C**) Venn diagram of the up-regulated transcripts in response to *cco-1* RNAi treatment in young (day 1) and aged (day 11) worms. (**D**) Functional clustering of the 279 transcripts as indicated in (C). (**E**) The representative genes that are differentially expressed upon *cco-1* RNAi in young and aged worms (log_2_FC relatively to control RNAi at day 1). FC, fold change. Genes up-regulated by *cco-1* RNAi treatment at both day 1 and day 11 are highlighted in red; genes up-regulated at day 11, but not at day 1, are highlighted in blue. (**F**) Functional clustering of the 2901 transcripts as indicated in (C). (**G**) Induction of GFP expression upon *cco-1*, *cyc-1*, *nuo-2*, *atp-3*, *mrps-5*, or *cts-1* RNAi in *cpr-5p::gfp* worms. DIC, differential interference contrast. Scale bar, 0.3 mm.

To systematically investigate how mild mitochondrial stress promotes longevity, we examined the temporal transcriptomic signatures of *C. elegans* treated with either control or mitochondrial respiration inhibition by *cco-1* RNAi (*8*), harvested at different adult stages, from young (day 1) to old (day 11) every other day (Fig. 1A; fig. S1, A and B; and table S1). In line with aging-related transcriptomic changes found in murine tissues (*40*, *41*), processes including “DNA repair,” “nucleotide excision repair,” “DNA helicase activity,” “ciliary part,” and “macroautophagy” were generally up-regulated with age (Fig. 1B and fig. S1, C to E), whereas processes related to “extracellular region part,” “innate immune response,” “lysosome,” “proteolysis,” “carbon metabolism,” “lipid metabolic process,” and “oxidative phosphorylation” were down-regulated (Fig. 1B and fig. S1, C to E). The aging-associated DEGs identified by comparing aged (days 9 and 11) and young (day 1) worms overlapped with 70.9% of the 1034 genes reported in a previous study using DNA microarrays (fig. S1C) (*42*), supporting the reliability of our transcriptome dataset. Binarized transcriptomic (BiT) aging Clock analysis (*43*) of the transcriptome data validated that *cco-1* RNAi worms were biologically younger (fig. S1F).

Next, we compared gene expression profiles between control and mitochondrial-stressed (*cco-1* RNAi) animals. Most of the aging-associated pathways showed almost identical footprints between the two groups (Fig. 1B and fig. S1E). In contrast, for the “lysosome” gene set, which significantly down-regulated in aged control animals [false discovery rate (FDR) = 2.6 × 10^−4^, day 11 versus day 1], was much less affected in *cco-1* RNAi worms (FDR = 0.2669, day 11 versus day 1) (Fig. 1B and fig. S1G). A similar trend was also found for the gene set of “endopeptidase activity,” a subset of the “proteolysis” (Fig. 1B). Further analysis of the *cco-1* RNAi–induced DEGs revealed that 279 transcripts demonstrated increased expression (Log_2_FC > 0.5, adjusted *P* < 0.05) in both day 1 and day 11 worms (Fig. 1C), and were enriched for processes including “lysosome” (e.g., *cpr-5*), “proteolysis” (e.g., *asp-1*), “innate immune response” (e.g., *clec-76*), and “metabolic pathways” (e.g., *sodh-1*) (Fig. 1, D and E). These gene ontology (GO) terms were also enriched for the 2901 transcripts exclusively up-regulated in aged *cco-1* RNAi worms, though with different ranks (Fig. 1, C and F). The expression of two mitochondrial chaperones, *hsp-6* and *dnj-10*, markers of the UPR^mt^ (*22*), was transiently up-regulated in *cco-1* RNAi worms only at adult day 1, but overall unchanged upon normal aging (at least until adult day 11) (Fig. 1E).

To determine whether the induction of lysosomal transcripts represents a general response downstream of diverse stress pathways, we exposed multiple reporter strains to a panel of classical stressors, including endoplasmic reticulum (ER) stress inducers (tunicamycin and *hsp-3* RNAi), cytosolic stress (heat-shock), and oxidative stress (*daf-2* RNAi). We found that none of these treatments effectively altered green fluorescent protein (GFP) intensity in a *cpr-5p::gfp* reporter strain (*38*), which expresses a fluorescent reporter for *cpr-5*, a *C. elegans* ortholog of human lysosomal protease cathepsin B (*44*) (fig. S2, A to C). In contrast, a variety of mitochondrial perturbations, including the RNAi of *cco-1* (ETC complex IV), *cyc-1* (ETC complex III), *nuo-2* (ETC complex I), *atp-3* (ETC complex V), *mrps-5* (mitochondrial ribosome small subunit), and *cts-1* (TCA cycle enzyme) (*8*–*11*), all robustly induced GFP expression in *cpr-5p:gfp* worms (Fig. 1G). Consistently, a panel of lysosomal proteases including *cpr-5*, *cpr-8*, *asp-1*, *asp-10*, *F32H5.1*, *F57F5.1*, and *ctsa-1*, was elevated in diverse conditions with mitochondrial respiration inhibition (fig. S2, D and E). These results demonstrate that activation of lysosome/proteolysis-related gene expression is a rather specific consequence downstream of mitochondrial stress.

To examine whether activation of the lysosomal reporter *cpr-5p::gfp* upon mitochondrial stress is restricted to a specific window during development, we initiated *cco-1* RNAi at different developmental stages. *Cpr-5p::gfp* was induced when *cco-1* RNAi was started from eggs up to the L4 stage, but not when started at the young adult or full adult stage (fig. S2F). This temporal pattern is similar to that of the canonical UPR^mt^ reporter *hsp-6p::gfp* (*20*). However, unlike *hsp-6p::gfp*, *cpr-5p::gfp* remained responsive when RNAi was initiated at L4, suggesting a slightly broader or distinct activation window.

### GATA TF ELT-2 links mitochondrial stress to lysosomal gene activation

**Fig. 2.**
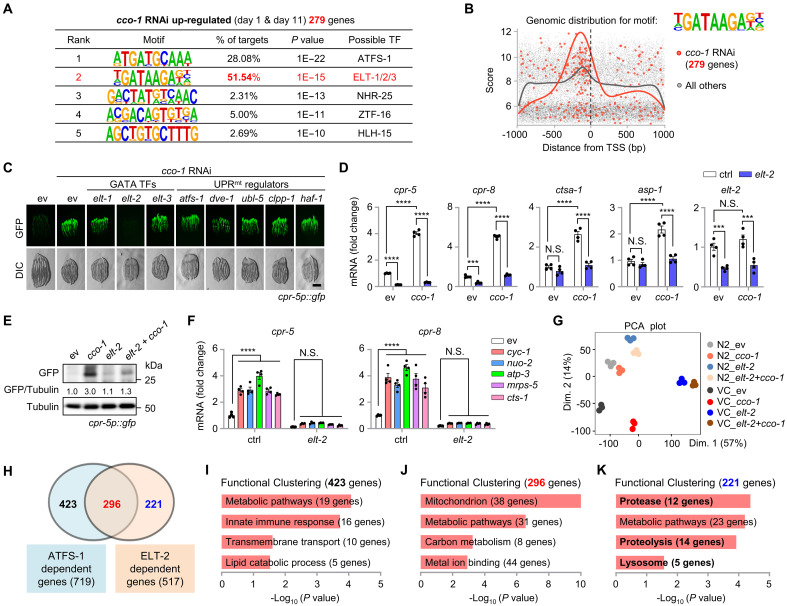
ELT-2 links mitochondrial stress to increased expression of lysosomal genes. (**A**) The top five enriched motifs for promoters of the 279 up-regulated genes upon *cco-1* RNAi at day 1 and day 11, by using hypergeometric optimization of motif enrichment (HOMER) and ranked on the basis of the *P* values. *P* value was derived from HOMER (one-sided hypergeometric test). (**B**) The genomic distribution of the motif hits at the promoters of 279 up-regulated genes upon *cco-1* RNAi, and all other genes. Scores are assigned on the basis of the position weight matrix (PWM) of the motif. Lines indicate the density of the motif hits. TSS, transcription start site. (**C**) RNAi of *elt-2* attenuated the GFP expression of *cpr-5p::gfp* worms induced by *cco-1* RNAi. Scale bar, 0.3 mm. (**D** and **E**) qRT-PCR analysis (*n* = 4 biologically independent samples) (D) and Western blots (E) of *cpr-5p::gfp* worms treated with control, *cco-1*, and/or *elt-2* RNAi. (**F**) qRT-PCR analysis (*n* = 4 biologically independent samples) of transcripts in worms fed with control, *cyc-1*, *nuo-2*, *atp-3*, *mrps-5*, or *cts-1* RNAi, with or without *elt-2* RNAi. (**G**) PCA plot of the RNA-seq profiles of worms treated with the indicated RNAi. (**H**) Venn diagram of ATFS-1–dependent and ELT-2–dependent *cco-1* RNAi–induced genes, according to the RNA-seq data. (**I** to **K**) Functional clustering of the 423 (I), 296 (J), and 221 (K) genes as indicated in (H). Error bars denote SEM. Statistical analysis was performed by ANOVA followed by Tukey post hoc test (****P < 0.0001 and ***P < 0.001; N.S., not significant).

Next, we sought to identify which TFs are responsible for the induction of these lysosomal/proteolytic genes. By analyzing the promoter sequences of the 279 genes up-regulated upon *cco-1* RNAi (Fig. 2A), an ATFS-1 binding motif emerged as the top hit (*P* = 1 × 10^−22^, one-sided hypergeometric test), in line with its established role in the UPR^mt^ branch of mitochondrial stress response (MSR) (*22*). The second most enriched motif, “TGATAAGAGT,” predicted to be recognized by GATA family TFs ELT-1/2/3 (*P* = 1 × 10^−15^, one-sided hypergeometric test), was found in over half (51.54%) of these MSR genes (Fig. 2A and table S1). A similar GATA-containing motif was furthermore identified as the top-ranked motif by analyzing the promoters of the 2901 *cco-1* RNAi–up-regulated transcripts in aged worms (day 11) (*P* = 1 × 10^−69^, one-sided hypergeometric test) (fig. S3A). Notably, both the two GATA-containing motifs were preferentially enriched at ∼100 bp upstream of the transcription start sites (TSSs), highlighting a plausible key role of these putative GATA motifs in regulating gene transactivation (Fig. 2B and fig. S3B). To investigate whether ELT-2 also functions as a transcriptional repressor under mitochondrial stress, we performed promoter motif analysis on genes down-regulated upon *cco-1* RNAi. In contrast to the up-regulated genes, the down-regulated gene sets did not show high enrichment of the canonical GATA motif recognized by ELT-2 (fig. S3, C to E). Instead, the most highly enriched motifs corresponded to binding sites for other TFs, including NHR-5, EGL-5, CND-1, CEH-18, and CES-1, suggesting that ELT-2 primarily acts as an activator rather than a repressor in this context.

We then conducted an RNAi-based screen covering all 14 known GATA-family TFs in *C. elegans* (*42*) and classical MSR regulators, we found that RNAi of *elt-2* strongly suppressed the activation of *cpr-5p::gfp* induced by *cco-1* RNAi (Fig. 2, C and D, and fig. S3F). In contrast, RNAi of the canonical UPR^mt^ regulators (*17*), or *hlh-30* (the ortholog of mammalian TFEB) (*45*), did not affect GFP expression in mitochondrial-stressed *cpr-5p:gfp* worms (Fig. 2C and fig. S3F). Endogenous expression of representative lysosomal proteases including *cpr-5* and *cpr-8* were also abolished by *elt-2* RNAi upon *cco-1* silencing or different kinds of mitochondrial perturbations (Fig. 2, E and F). To further clarify the roles of ATFS-1 and ELT-2 in MSR regulation, we compared transcriptional profiles of wild-type and the *atfs-1(gk3094)* loss-of-function mutant subjected to *cco-1* and/or *elt-2* RNAi (Fig. 2G and table S2). Knockdown of *cco-1* resulted in the up-regulation of 1798 transcripts, among which 517 were ELT-2–dependent and 719 required ATFS-1 for induction (Fig. 2H). In line with previous studies (*17*, *22*), ATFS-1–regulated MSR transcripts (including 423 “ATFS-1 only” and 296 “ATFS-1 and ELT-2 commonly-regulated”), were enriched for typical UPR^mt^-targeting pathways such as “mitochondrion,” “carbon metabolism,” and “innate immune response” (Fig. 2, I and J). Within the 517 MSR transcripts regulated by ELT-2, 221 (42.7%) were specifically dependent on ELT-2 (Fig. 2H), and were enriched for pathways related to “protease,” “metabolic pathways,” “proteolysis,” and “lysosomes” (Fig. 2K). Quantitative reverse transcription polymerase chain reaction (qRT-PCR) analysis validated that *cco-1* silencing–induced up-regulation of lysosomal protease genes, including *cpr-5*, *cpr-8*, *asp-1*, and *nep-17*, exclusively required ELT-2, but not ATFS-1 (fig. S3G). In addition, loss of the mitophagy regulators *dct-1* or *pdr-1* did not impair the up-regulation of ELT-2–targeting lysosomal protease genes (e.g., *cpr-5*, *cpr-8*, *asp-1*, and *nep-22*) upon *cco-1* RNAi (fig. S3H). Collectively, these findings suggest that ELT-2 predominately mediates the lysosomal branch of the MSR, operating in parallel with the canonical UPR^mt^ and mitophagy pathways.

### ELT-2 is essential for mitochondrial respiration inhibition–induced lifespan extension

**Fig. 3.**
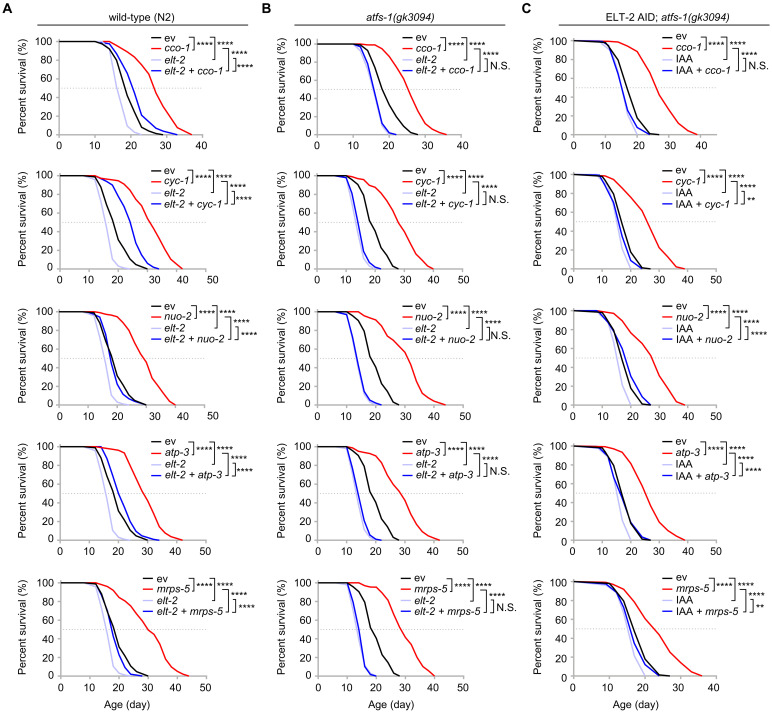
A crucial role of ELT-2 in mitochondrial respiration inhibition–induced lifespan extension. (**A** and **B**), Survival of wild-type (N2) (A) and *atfs-1(gk3094)* (B) worms fed with control (ev), *cco-1*, *cyc-1*, *nuo-2*, *atp-3*, *mrps-5*, and/or *elt-2* RNAi. (**C**) Survival of ELT-2 AID (*elt-2::Degron::mNeonGreen;eft-3p::TIR1::mRuby*); *atfs-1(gk3094)* worms treated with control, *cco-1* RNAi and/or 0.1 mM indole-3-acetic acid (IAA). Statistical analysis was performed by log-rank test (**P < 0.01 and ****P < 0.0001; N.S., not significant). Statistical data for lifespan can be found in table S3.

We next asked whether ELT-2 contributes to the lifespan extension induced by mitochondrial respiration inhibition. Knockdown of *elt-2* substantially suppressed the longevity caused by *cco-1* RNAi (Fig. 3A). However, a mild but significant lifespan extension was still observed upon *elt-2/cco-1* double RNAi treatment compared to *elt-2* RNAi alone (Fig. 3A). We hypothesize that ATFS-1-mediated MSR may contribute to the remaining longevity response while *elt-2* was silenced. In support of such an assumption, *cco-1* RNAi–induced longevity was completely abolished by *elt-2* RNAi in *atfs-1(gk3094)* loss-of-function mutants (Fig. 3B). Similar results were also observed with other mitochondrial respiration suppressors, including *cyc-1*, *nuo-2*, *atp-3*, and *mrps-5* RNAi, though with varied residual lifespan extension after *elt-2* silencing (Fig. 3, A and B).

As another approach to disrupt *elt-2* function, we used the auxin-induced degradation (AID) system for ELT-2 degradation (*46*). Using the CRISPR-Cas9 technology, we generated an *elt-2::degron::mNeonGreen* knock-in strain expressing somatic *eft-3p::TIR1-mRuby* (ELT-2 AID) (*38*), and then introduced it into an *atfs-1(gk3094)* background. Exposure of the ELT-2 AID; *atfs-1(gk3094)* worms with 0.1 mM natural auxin indole-3-acetic acid (IAA) effectively reduced ELT-2 protein levels (fig. S4A), and almost completely blocked mitochondrial respiration inhibition–induced lysosomal protease gene expression and lifespan extension (Fig. 3C and fig. S4B), phenocopying the *elt-2* RNAi results (Figs. 2E and 3B).

To genetically validate the role of ELT-2 in mitochondrial-stress induced lysosomal protease gene expression, we used the *JM69*(*elt-2^+/−^*) strain, which carries only one functional copy of *elt-2*. In wild-type animals, *cco-1* RNAi robustly induced the lysosomal protease genes *cpr-5*, *cpr-8*, *ctsa-1*, and *asp-10* (fig. S5, A and B). In contrast, this induction was attenuated in *JM69*(*elt-2^+/−^*) worms. These results demonstrate that full activation of the ELT-2–mediated lysosomal transcriptional program under mitochondrial stress likely requires both functional *elt-2* alleles, highlighting that ELT-2 may act as a dose-dependent master regulator of this adaptive response.

**Fig. 4.**
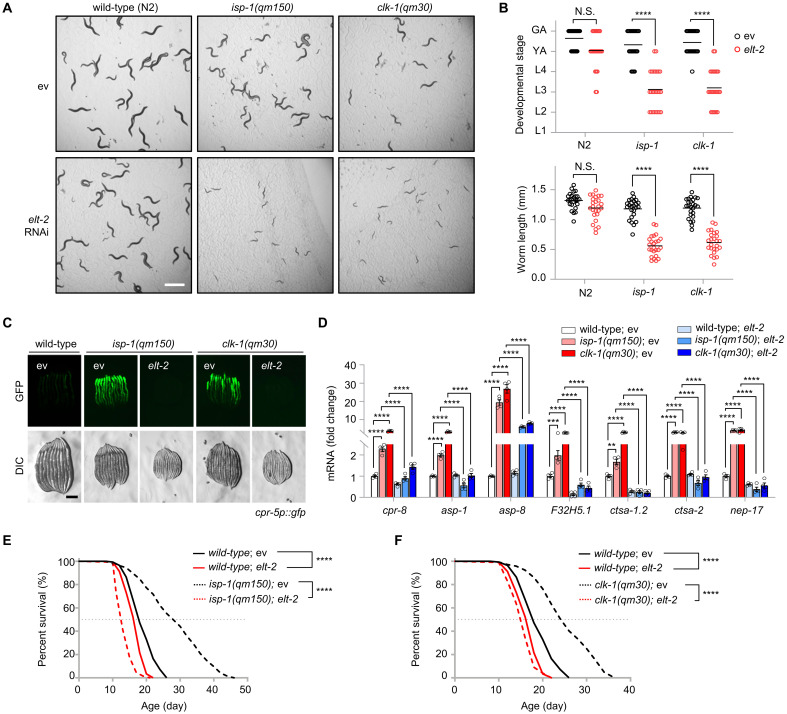
Long-lived mitochondrial respiration defective worms highly rely on ELT-2 for their synthetic growth, lysosomal proteases expression and longevity. (**A** and **B**) Representative photos (A) of wild-type (N2), *isp-1(qm150)* and *clk-1(qm30)* worms fed with control (ev), or *elt-2* (10%) RNAi since egg stage. Scale bar, 1 mm. The developmental stage (top) and body length (bottom) of the F1 progeny were quantified (B) at day 4 after hatching (*n* = 25 worms for each condition). GA, gravid adult; L1-L4, larval stages 1 to 4; YA, young adult. (**C**) RNAi of *elt-2* (10%) attenuated the GFP expression of *cpr-5p::gfp* worms in *clk-1(qm30)* and *isp-1(qm150)* mutants. Scale bar, 0.3 mm. (**D**) qRT-PCR analysis (*n* = 4 biologically independent samples) of wild-type (N2), *isp-1(qm150)* and *clk-1(qm30)* worms treated with control (ev) or *elt-2* (10%) RNAi. (**E** and **F**) Survival of wild-type (N2), *isp-1(qm150)* and *clk-1(qm30)* mutant worms fed with control or *elt-2* (10%) RNAi. Error bars denote SEM. Statistical analysis was performed by ANOVA followed by Tukey post hoc test [(B) and (D)] or log-rank test [(E) and (F)] (**P < 0.01, ***P < 0.001, and ****P < 0.0001; N.S., not significant). Statistical data for lifespan can be found in table S3.

To further validate the role of ELT-2 in inter-organelle communication upon mitochondrial stress and mitohormesis-associated longevity, we raised wild-type (N2) worms and the long-lived mitochondrial respiration mutants *isp-1(qm150)* and *clk-1(qm30)* (*26*, *47*), on control or *elt-2* RNAi. Compared with animals fed with control RNAi, *elt-2* RNAi even at a dilution of 10% led to severe synthetic growth defects of both the mitochondrial ETC mutants, whereas the development of wild-type worms was only slightly delayed (Fig. 4, A and B). Likewise, *cco-1* RNAi treatment results in a synergistic reduction in worm size when combined with *elt-2* RNAi, which becomes more pronounced in the background of *atfs-1(gk3094)* loss-of-function mutant (fig. S5, C and D). Consistently, representative lysosomal protease genes (i.e., *cpr-8*, *asp-1*, *asp-8*, *F32H5.1*, *ctsa-1.2*, *ctsa-2*, and *nep-17*) were robustly induced in *isp-1(qm150)* and *clk-1(qm30)* mutants (*39*), and in an ELT-2–dependent manner (Fig. 4, C and D). Notably, *elt-2* silencing not only abrogated the long-lived phenotype of these mitochondrial mutants but also shortened their lifespans to below that of wild-type animals (Fig. 4, E and F). Together, these results demonstrate that ELT-2 is essential for mediating mitochondrial inhibition-induced lifespan extension, and that mitochondrial respiration-defective mutants strongly rely on ELT-2 to maintain normal growth and longevity.

### Increased ELT-2 expression upon mitochondrial stress mediates transactivation of lysosomal genes via specific GATA motif and the ELT-2–R249 residue

**Fig. 5.**
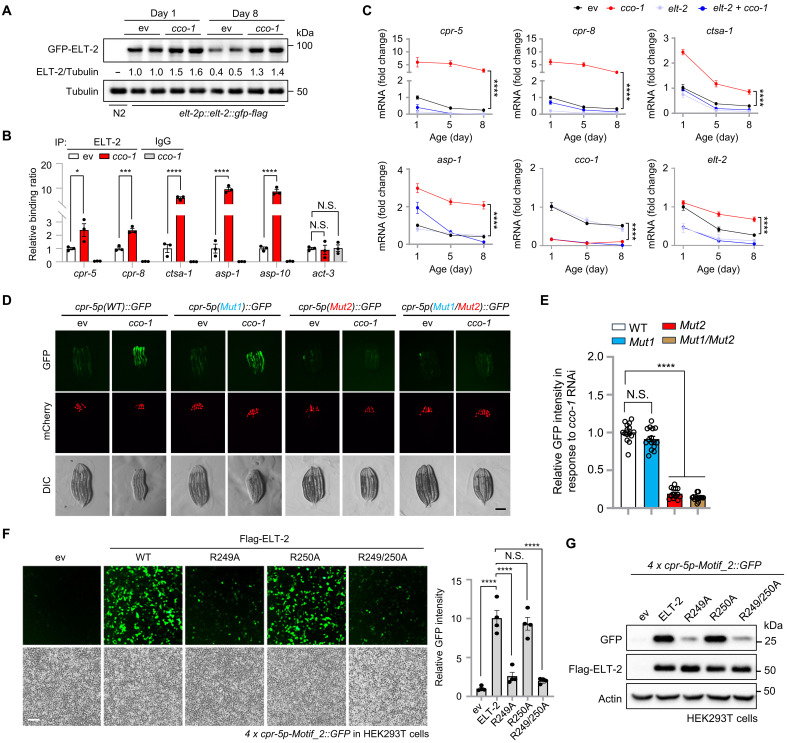
Increased ELT-2 expression upon mitochondrial stress mediates transactivation of lysosomal genes via specific GATA motif and the ELT-2–R249 residue. (**A**) Western blots of *elt-2p::elt-2::gfp-flag* worms fed with control or *cco-1* RNAi, and harvested at adulthood day 1 and day 8. (**B**) ChIP-qPCR analysis (*n* = 3 biologically independent samples) of *elt-2p::elt-2::gfp-flag* worms fed with control or *cco-1* RNAi. IP, immunoprecipitation. (**C**) The mRNA levels of indicated genes (*n* = 4 biologically independent samples) of worms fed with control, *cco-1* and/or *elt-2* RNAi, collected at different ages. (**D**) Representative images of the transgenic worms carrying the WT or mutated *cpr-5* promoter driving GFP reporter constructs fed with control or *cco-1* RNAi. Plasmids expressing *myo-2p::mCherry* (to mark the pharynx with red fluorescent) were used as coinjection markers to validate successfully generation of each strain. Scale bar, 0.2 mm. (**E**) Quantification of GFP fluorescence intensity of worms fed with *cco-1* RNAi as indicated in (D) (*n* = 15 independent worm images for each condition). (**F**) GFP fluorescence images of HEK293T cells transfected with a GFP reporter driven by four tandem repeats of the *cpr-5p-Motif_2* (5′-ACTGATAAGA-3′) and cotransfected with empty vector (ev) control, wild-type (WT) ELT-2 or ELT-2 mutations. The relative GFP intensity was quantified (*n* = 4 independent biological replicates). Scale bar, 0.2 mm. (**G**) Western blots of cells as indicated in (F). Error bars denote SEM. Statistical analysis was performed by ANOVA followed by Tukey post-hoc test (*P < 0.05, ***P < 0.001, and ****P < 0.0001; N.S., not significant).

To understand how ELT-2 mediates the longevity induced by mitochondrial respiration inhibition, we first examined its expression dynamics during aging and under mitochondrial stress. Exogenously expressed ELT-2::GFP localized predominantly to intestinal nuclei (*38*, *48*), as visualized by costaining with the blue-fluorescent DNA dye 4′,6-diamidino-2-phenylindole (DAPI), and exhibited an age-dependent decline (fig. S6A). In contrast, *cco-1* RNAi not only elevated ELT-2 expression but also sustained it at high levels into late adulthood (up to day 8) (Fig. 5A and fig. S6A). Similar results were also observed by checking the endogenous ELT-2 expression in an *elt-2::degron::mNeonGreen* knock-in worm strain coexpressing mCherry-tagged histone (fig. S6B). Accordingly, the mRNA level of *elt-2*, but not other nuclear-localized MSR regulators, such as *atfs-1* (*22*), *dve-1* (*49*), *ubl-5* (*50*), *hda-1* (*51*, *52*), and *cbp-1* (*53*), declined with age, and this decline was markedly attenuated under mitochondrial stress (fig. S6C).

Next, by applying chromatin immunoprecipitation coupled with quantitative PCR (ChIP-qPCR) analysis, we detected strong enrichment of ELT-2 at the promoters of lysosomal protease genes (e.g., *cpr-5*, *cpr-8*, *ctsa-1*, *asp-1*, and *asp-10*), which was further enhanced following *cco-1* RNAi (Fig. 5B), suggesting that mitochondrial stress promotes direct transcriptional control of these lysosomal proteases by ELT-2. Consistently, the up-regulation of lysosomal proteases in aged animals induced by *cco-1* RNAi was fully suppressed by *elt-2* knockdown (Fig. 5C).

To functionally validate the reciprocal interaction between ELT-2 and lysosomal gene promoters, we first generated transgenic worms carrying mutations of two predicted ELT-2 binding motifs at positions −643 bp (*Motif_1*) and −61 bp (*Motif_2*) upstream of the *cpr-5* TSS (fig. S7A). Mutation of *Motif_2*, but not *Motif_1*, almost completely abolished *cpr-5p::gfp* induction upon *cco-1* RNAi (Fig. 5, D and E). ELT-2 shares a strong conservation with human GATA family proteins (*54*), particularly within the DNA binding zinc finger domain harboring two adjacent positively charged arginine residues (R249 and R250) (fig. S7B). To test the functional importance of these residues, we generated a GFP reporter driven by a 70-bp promoter that contains four tandem repeats of the *cpr-5p-Motif_2* (5′-ACTGATAAGA-3′). Cotransfection of this reporter with wild-type or R250A-ELT-2 robustly activated GFP expression in human HEK293T cells, whereas ELT-2 mutants carrying the R249A mutation exhibited markedly reduced reporter activity (Fig. 5, F and G). These results highlight a direct regulatory mechanism of ELT-2 in mediating transcriptional activation of lysosomal genes via the R249 residue and specific GATA-containing DNA motif.

To further gain structural insights on how ELT-2 recognizes the GATA motifs in lysosomal gene promoters, we performed molecular docking to model the interaction between ELT-2 and the *cpr-5p-Motif_2* DNA sequence. We found that the R249 residue of wild-type ELT-2 forms two putative hydrogen bonds/electrostatic interactions with the DNA, with distances of 3.6 and 2.9 Å, respectively; R250 interacts with DT-14 and DT-13 (2.8 and 3.2 Å) (fig. S7C). In contrast, alanine substitution in R249 (ELT-2–R249A mutant) completely eliminates the side-chain interactions provided by R249; while the R250-DT-13 interaction is partially retained (3.3 Å), the R250-DT-14 interaction is also lost (fig. S7C). Collectively, these docking models provide direct structural evidence that R249 acts as a key residue for ELT-2 binding to GATA motifs that are enriched in certain lysosomal gene promoters.

### Mitochondrial stress mitigates age-related lysosomal deacidification and enhances lysosomal activity

**Fig. 6.**
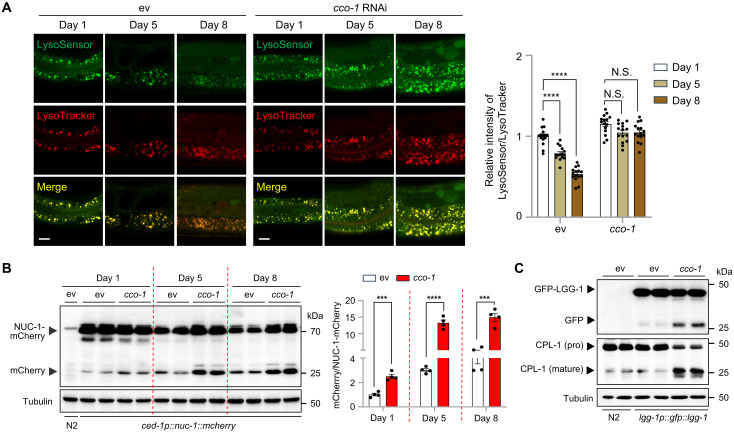
Mitochondrial stress mitigates age-related lysosomal deacidification and enhances lysosomal activity. (**A**) Confocal fluorescent images and quantification (*n* = 15 individual worms for each condition) of LysoSensor Green and LysoTracker staining of the intestine of worms fed with control (ev) or *cco-1* RNAi, analyzed at days 1, 5, and 8 of adulthood. Pictures in the same channels were taken at the same settings. Scale bars, 10 μm. (**B**) Western blots of wild-type (N2) and *ced-1p::nuc-1::mCherry* worms fed with control or *cco-1* RNAi and analyzed at days 1, 5, and 8 of adulthood. The mCherry versus NUC-1-mCherry was quantified (*n* = 4 independent experiments). (**C**) Western blots of wild-type (N2) or *lgg-1p::gfp::lgg-1* worms fed with control or *cco-1* RNAi, analyzed at day 5. Error bars denote SEM. Statistical analysis was performed by ANOVA followed by Tukey post hoc test (***P < 0.001 and ****P < 0.0001; N.S., not significant).

To directly reveal the impact of mitochondrial respiration inhibition on lysosomal function, we first checked lysosomal acidification by costaining worms with LysoSensor Green and LysoTracker Red (*39*, *55*). LysoTracker is less sensitive to increased pH than LysoSensor and was used as a dye intake control (*39*, *55*). In control worms, the LysoSensor/LysoTracker signal ratio decreased as animals aged (Fig. 6A). By contrast, *cco-1* RNAi–treated animals maintained a notably higher signal ratio even at day 8 (Fig. 6A), suggesting that mitochondrial stress preserves lysosomal acidification during aging. Next, we evaluated lysosomal degradative capacity using a NUC-1::mCherry fusion protein (*56*). When delivered to lysosomes, mCherry is cleaved from this NUC-1-mCherry fusion protein by cathepsins, and the extent of cleavage can be visualized to indicate the degradation activity of lysosomes. Compared with controls, *cco-1* RNAi–treated worms exhibited enhanced mCherry cleavage at both young and old ages, with the most substantial difference observed at day 5 (Fig. 6B). Of note, cleaved mCherry progressively accumulated with age due to its resistance to lysosomal degradation (*57*). Likewise, using the autophagic reporter *lgg-1p::gfp::lgg-1*, we observed a trend of reduced full-length GFP–LGG-1 and increased processed GFP in *cco-1* RNAi worms (Fig. 6C), indicating elevated autophagic-lysosomal activity. Consistently, maturation of the lysosomal protease cathepsin L (CPL-1), which is synthesized as an inactive pro form and converted to the active mature form through proteolytic removal of the pro-domain in lysosomes (*39*, *58*), was strongly enhanced under *cco-1* RNAi (Fig. 6C). Collectively, these results suggest that mitochondrial respiration inhibition induced by *cco-1* RNAi counteracts age-associated lysosomal deacidification and enhances overall lysosomal activity in *C. elegans*.

### Enhanced lysosomal proteostasis mediates mitochondrial stress–induced polyQ clearance and lifespan extension

**Fig. 7.**
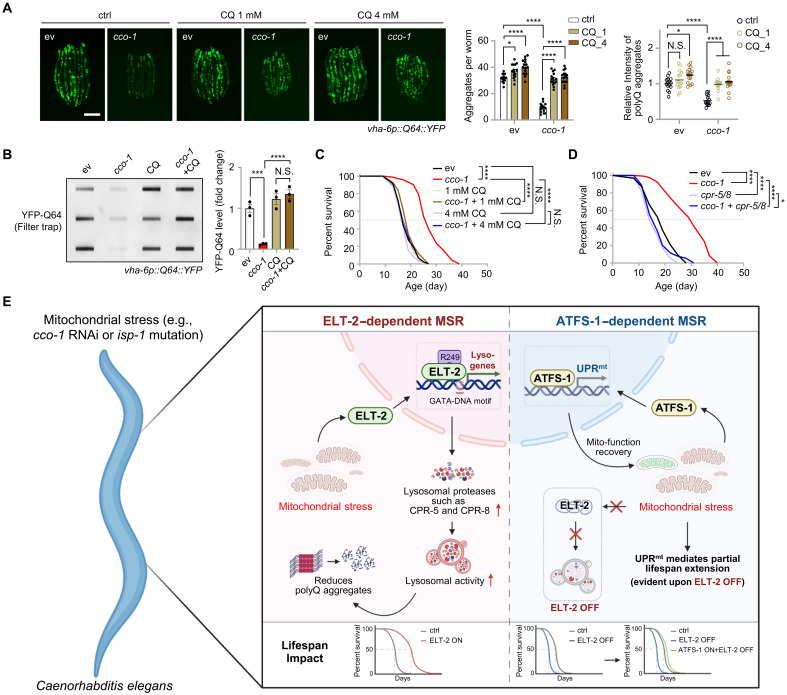
Enhanced lysosomal proteostasis mediates mitochondrial stress–induced polyQ aggregation clearance and lifespan extension. (**A**) Worms expressing *vha-6p::Q64::YFP* were treated with control or *cco-1* RNAi, and/or 1 to 4 mM chloroquine (CQ), analyzed at day 5. Scale bar, 0.3 mm. The number and average intensity of polyQ aggregates (*n* = 15 individual worms for each condition) were quantified. (**B**) Filter trap analysis (*n* = 3 biologically independent samples) of *vha-6p::Q64::YFP* worms fed with control or *cco-1* RNAi and/or 1 mM CQ at adult day 5. (**C**) Survival of worms fed with control or *cco-1* RNAi, and/or 1 to 4 mM CQ. (**D**) Survival of worms fed with control or *cco-1* RNAi (50%), and/or *cpr-5* + *cpr-8* RNAi (each occupied 25%). (**E**) A model depicts the role of ELT-2– and ATFS-1–mediated mitochondria stress response in orchestrating lysosomal and mitochondrial homeostasis, coordinating cellular proteostasis and organismal longevity. Created in BioRender. Yang, R. (2026) https://BioRender.com/hw6yq9e. Error bars denote SEM. Statistical analysis was performed by ANOVA followed by Tukey post hoc test [(A) and (B)] or log-rank test [(C) and (D)] (*P < 0.05, ***P < 0.001, and ****P < 0.0001; N.S., not significant). Statistical data for lifespan can be found in table S3.

During the aging process, the accumulation of misfolded or aggregated proteins typically increases, contributing to the pathogenesis of neurodegenerative diseases (*59*). We thus asked whether mitochondrial stress–induced activation of lysosomal-associated genes and the consequent increase in lysosomal activity could help alleviate proteotoxicity in vivo. In a worm HD model (GF83) that expresses polyQ-expanded (Q64) Huntingtin-like proteins in the intestine (*60*), *cco-1* RNAi robustly reduced the aging-dependent accumulation of polyQ aggregates (fig. S8, A and B). This protective effect was abolished by treatment with the lysosomal inhibitor chloroquine (CQ) at either 1 mM or 4 mM (Fig. 7, A and B, and fig. S8C), indicating a lysosome-dependent clearance mechanism. Consistently, reductions in the number of polyQ aggregates were also found in *vha-6p::Q64::YFP* worms treated with other mitochondrial perturbations (e.g., *cyc-1*, *nuo-2*, *atp-3*, and *mrps-5* RNAi) (fig. S8D). To determine whether the mito-stress–ELT-2–lysosome pathway broadly enhances proteostasis, we tested two additional aggregation-prone protein models: the AM725 strain expressing SOD-1::YFP [a model of amyotrophic lateral sclerosis (ALS)] and the NL5901 strain expressing α-synuclein::YFP [a model of Parkinson’s disease (PD)]. RNAi of *cco-1* remarkably reduced aging-associated accumulation of both SOD-1 and α-synuclein aggregates (fig. S9, A to D). Of note, the monomeric and submonomeric forms of α-synuclein were also diminished upon *cco-1* RNAi (fig. S9E). These results demonstrate that the beneficial effect of mild mitochondrial stress on proteostasis may be not limited to polyQ, but also to other aggregation models, including PD and ALS.

To further identify which lysosomal proteases are responsible for such beneficial effect, we performed another screen with RNAi targeting each of the eight cathepsins (i.e., *cpr-5*, *cpr-8*, *F32F5.1*, *F57F5.1*, *cpr-3*, *ctsa-1*, *ctsa-2*, and *W07B8.4*), three aspartic endopeptidases (i.e., *asp-1*, *asp-8*, and *asp-10*), and two metallo-endopeptidase (*nep-17* and *nep-22*) that are up-regulated by *cco-1* RNAi (table S1). RNAi of *cpr-5* or *cpr-8*, but not other proteases, partially suppressed the reduction in polyQ aggregates induced by *cco-1* RNAi (fig. S10, A to C). Notably, both CQ treatment and *cpr-5* or *cpr-8* loss-of-function (achieved via either RNAi or knockout) robustly suppressed the longevity induced by *cco-1* RNAi (Fig. 7, C and D, and fig. S10, D and E). Together, these results suggest an essential role of lysosomal function, as well as the cathepsin proteases CPR-5 and CPR-8, in mediating mitochondrial stress–induced clearance of polyQ protein aggregation and lifespan extension.

## DISCUSSION

The cross-talks between mitochondria and lysosomes are pleiotropic and can be achieved by multiple forms, including at least via the physical contact sites, metabolites, calcium, proteotoxic stress, ROS, mitochondria-derived vesicles, and mitophagy (*1*, *61*). While severe disruption of mitochondria by genetic deletion of the *mitochondrial transcription factor A* (*Tfam*) disrupts the endolysosomal trafficking pathways and impairs lysosome function in CD4^+^ T lymphocytes (*62*), multiple adaptation mechanisms have evolved to sustain organellar homoeostasis as well. For example, an AMPK-FNIP1-TFEB axis is activated to promote lysosomal and mitochondrial biogenesis upon mitochondrial damage (*63*). Metabolic restructuring caused by mitochondrial stress could also trigger changes in gene expression coordinated by both the mitochondrial and cytosolic UPRs, protecting cells from disease-associated proteins (*64*). Intriguingly, a recent study demonstrated that induction of specific lysosomal signaling mediated by *lipl-4* overexpression increases mitochondrial β-oxidation to reduce lipid storage and promote longevity (*65*). However, whether and how lysosomal adaptations might contribute to the beneficial effects of mitochondrial stress, or participate in mitohormesis at cellular and organismal levels have remained largely unclear.

In this study, by systematically analyzing the aging-associated transcripts up-regulated in response to mitochondrial respiration inhibition in *C. elegans*, we identify an ELT-2–mediated lysosomal branch of MSR as a central mechanism underlying the beneficial effect of mitohormesis. We demonstrate that mitochondrial inhibition, achieved through either ETC gene inhibition or mutation (e.g., *cco-1* RNAi or *isp-1* mutation), activates a sustained transcriptional program of lysosomal and proteolytic genes, which is predominately mediated by the GATA TF ELT-2. This ELT-2–dependent adaptive pathway preserves lysosomal acidification and degradative capacity during aging, reduces protein aggregation (as exemplified by clearance of polyQ aggregates), and is essential for the longevity conferred by mitochondrial stress. Meanwhile, ATFS-1–dependent MSR, the UPR^mt^, mainly contributes to mitochondrial functional recovery by mediating the induction of transcripts involved in mitochondrial and metabolic pathways (*22*). Despite that ATFS-1–mediated UPR^mt^ activation alone is neither strictly necessary nor sufficient for lifespan extension in certain contexts (*23*–*25*), its role in longevity becomes evident when *elt-2* is silenced. Collectively, these results highlight two distinct yet complementary branches of the MSR that orchestrating mitochondrial and lysosomal homeostasis, coordinating organismal fitness and lifespan extension (Fig. 7E).

How ELT-2 senses the mitochondrial stress remains an important direction for future work. One possibility is that ELT-2 itself is a downstream target that is activated in response to mitochondrial perturbations, as evidenced by increased ELT-2 expression after *cco-1* silencing (Fig. 5, A and C). Changes in mitochondrial-derived signaling molecules, such as the ROS, may also acts as key messengers and modulate ELT-2–mediated transcription via the target of rapamycin signaling upon stresses in addition to hypoxia (*66*).

Our work positions ELT-2 as a central regulator of mitochondrial stress–induced lysosomal activation. Other canonical longevity factors such as DAF-16/FOXO and HLH-30/TFEB have also been shown to mediate the expression of certain lysosomal genes (*39*, *45*). Nevertheless, the specific lysosomal genes targeted by DAF-16 and ELT-2 seems rather different (*39*). Likewise, ELT-2 drives lysosomal gene induction independently of HLH-30 upon *cco-1* RNAi (fig. S3F). Thus, it is highly possible that ELT-2 defines a parallel, intestine-enriched pathway that operates largely independently of these global longevity regulators. Future studies employing tissue-specific rescue and epistasis analyses will help further clarify the precise hierarchical relationships among these TFs.

The transcriptional response induced by mitochondrial respiration inhibition partially overlaps with the program activated by disruption of intestinal luminal acidification (e.g., by *vha-6* RNAi) (*38*), suggesting that enhanced lysosomal activity may represent a shared adaptive strategy under distinct forms of cellular stresses (*39*, *67*, *68*). However, in contrast to mitochondrial perturbations, ER stress, heat shock, or oxidative stress did not activate the *cpr-5p::gfp* reporter (fig. S2, A to C). Moreover, ELT-2 protein level remains unchanged following *vha-6* RNAi (*38*), implying that mitochondrial inhibition likely engages a regulatory mechanism distinct from that triggered by intestinal acidification disruption or other stresses.

The reduction of age-associated polyQ aggregates following mitochondrial inhibition strongly depends on intact lysosomal function and the cathepsins CPR-5 and CPR-8 (Fig. 7, A and B, and fig. S10, A and B), highlighting a central role of mitochondria-lysosome communication pathways in cellular metabolism, stress signaling, and the pathogenesis of neurodegenerative diseases (*61*). In summary, we propose that the ELT-2–dependent MSR via the lysosomes constitutes a fundamental adaptive response that maintains proteostasis and extends lifespan in response to mitochondrial stress. Our work thereby not only redefines the mechanistic landscape of mitohormesis but also may open new avenues for developing interventions aimed at combating age-related proteotoxic neurodegenerative diseases and lifespan/healthspan extension through lysosomal enhancement.

## MATERIALS AND METHODS

### *C. elegans* strains

The N2 (Bristol) strain was used as the wild-type strain. VC3201 [*atfs-1(gk3094)*], MQ887 [*isp-1(qm150)*], MQ130 [*clk-1(qm30)*], RB1810 [*cpr-5(ok2344)*], RB2180 [*cpr-8(ok2956)*], JM69 [*dpy-5(e61), unc-13(e1091)/szT1(lon-2(e678)); elt-2(ca15)/szT1*], VC1024 [*pdr-1(gk448)*], MLC2543 [*dct-1(luc194)*], SJ4100 [*zcIs13 (hsp-6p::GFP)*], SJ4005 [*zcIs4 (hsp-4p::gfp)*], CL2070 [*dvIs70 [hsp-16.2p::gfp + rol-6(su1006)]*], KN259 [*huIs33 (sod-3p::gfp + rol-6(su1006))*], OP56[*gaEx290 [elt-2::TY1::EGFP::3xFLAG(92C12) + unc-119(+)]*], GF83 [*dgEx83 (pAMS56 vha-6p::Q64::YFP + rol-6(su1006) + pBluescript)*], AM140 [*rmIs132 (unc-54p::Q35::YFP)*], AM725 [*rmIs290 (unc-54p::Hsa-sod-1(127X)::YFP)*], NL5901 [*pkIs2386 (unc-54p::*α*-synuclein::YFP + unc-119(+))*], CA1200 [*ieSi57 (eft-3p::TIR1::mRuby::unc-54 3'UTR + Cbr-unc-119(+))*], and DA2123 (*adIs2122* [*lgg-1p::GFP::lgg-1* + *rol-6(su1006)*]) were provided by the Caenorhabditis Genetics Center (CGC; University of Minnesota). The XW19180 [*hsp-16.2p::nuc-1::pHTomato*] and XW5399 [*qxIs257 (ced-1p::nuc-1::mCherry + unc-76(+))*] strain was a kind gift from X. Wang (SUSTech). Strains previously generated by our laboratory are: TYL1 [*syuIs1 (cpr-5p::GFP + rol-6(+))*], TYL5 [*syu3 (elt-2p::elt-2::Degron::mNG)*], and TYL8 [*syu3 (elt-2p::elt-2::Degron::mNG)*; *ieSi57 (eft-3p::TIR1::mRuby::unc-54 3′UTR + Cbr-unc-119(+))*], which are all available from CGC (*38*).

The following strains were generated in the current study: TYL9 [*atfs-1(gk3094)*; *syu3 (elt-2p::elt-2::Degron::mNG)*; *ieSi57 (eft-3p::TIR1::mRuby::unc-54 3'UTR + Cbr-unc-119(+))*], TYL11 [*syuEx10 (cpr-5p(WT)::gfp + myo-2::mcherry + rol-6)*], TYL12 [*syuEx11 (cpr-5p(Mut1)::gfp + myo-2::mcherry + rol-6)*], TYL13 [*syuEx12(cpr-5p(Mut2)::gfp + myo-2::mcherry + rol-6)*], and TYL14 [*syuEx13 (cpr-5p(Mut1+Mut2)::gfp + myo-2::mcherry + rol-6)*]. All *C. elegans* were maintained on nematode growth media (NGM) with *Escherichia coli* OP50 as food under standard culture conditions at 20°C, unless specifically indicated.

To construct the *cpr-5p::gfp* reporter strains, the 1018 bp *cpr-5* promoter was amplified using the primers 5′-GAATTGACATGCACTCCGGC-3′ and 5′-AAGAATAGCGGAGAGCTTCC-3′. The amplified fragment was then cloned in-frame with eGFP into the pPD95.75 expression vector (Addgene, no. 184130), which was linearized via double digestion with SphI (catalog no. FD0604, Thermo Fisher Scientific) and KpnI (catalog no. FD0524, Thermo Fisher Scientific). The two ELT-2 binding motifs predicted within the *cpr-5* promoter sequence are: 5′-TTTCTTATCA-3′ (*Motif_1*, positions −634 to −643) and 5′-TGATAAGAAT-3′ (*Motif_2*, positions −61 to −70). Mutations of the promoter reporter (Mut1, Mut2, and Mut1 + Mut2) were introduced by replacing all bases to adenine (A) in the pPD95.75_*cpr-5p::gfp* plasmid. Transgenic lines were generated by injecting the constructs at 10 to 60 ng/μl, along with the coinjection marker pRF4 (*rol-6*) and *myo-2p::mcherry* at 40 ng/μl.

### RNA interference

For RNAi experiments, worms were fed with *E. coli* strains HT115(DE3) containing an L4440 empty vector (ev) or L4440 expressing double-strand RNAi. RNAi clones were obtained from either the Ahringer or Vidal library, verified by sequencing or tested for their knockdown efficiency by qRT-PCR before use. The RNAi clones for *elt-4*, *elt-6*, and *egl-27* were constructed by PCR amplification, ligated into the L4440 empty vector and transformed into *E. coli* HT115 competent cells, as described previously (*53*). Unless otherwise noted, RNAi treatments were initiated from the egg stage. For the RNAi experiments, NGM plates were further supplemented with carbenicillin (25 mg/ml) and 2 mM isopropyl β-d-1-thiogalactopyranoside to induce RNAi expression. RNAi bacteria were inoculated and cultured in lysogeny broth (LB) medium containing ampicillin (100 μg/ml; A610028, Sangon) overnight at 37°C. Mixed RNAi experiments were performed by mixing the bacterial cultures (normalized by optical density measured in optical density at 600 nm) before seeding.

### RNA extraction and RNA-seq analysis

Total RNA was extracted from synchronized *C. elegans* populations treated with different RNAi bacteria, and harvested at different time points. Approximately 1000 to 2000 worms per condition were harvested. Animals were washed three times with sterile M9 buffer to eliminate bacterial and environmental contaminants. Worm pellets were snap-frozen in liquid nitrogen and stored at −80°C until RNA extraction. For RNA isolation, 1 ml of TriPure Isolation Reagent (catalog no. 11667165001, Roche) was added to each worm pellet, repeated freezing and thawing by ways of a water bath (37°C) and liquid nitrogen. Phase separation was achieved via chloroform addition and centrifugation. The aqueous phase containing RNA was subsequently purified using the NucleoSpin RNA Clean-up XS kit (catalog no. 740955.250, Macherey-Nagel) to remove residual proteins, lipids, and genomic DNA. The concentration and purity of the extracted RNA were determined by NanoDrop spectrophotometry.

RNA sequencing (RNA-seq) was conducted by the Beijing Genomics Institute using the BGISEQ-500 platform or by the Novogene Bioinformatics Technology Co. Ltd. using the Illumina platforms with PE150 strategy. Only RNA samples with an RNA integrity number (RIN) ≥ 8.0 were used for library preparation. After end-repair, adaptor ligation, and PCR amplification, sequencing libraries were quantified and assessed for quality before clustering. Raw sequencing reads were processed to remove adapter sequences, low-quality reads (Phred score < 20), and reads containing >10% ambiguous nucleotides. High-quality reads were then mapped to the “*Caenorhabditis_elegans*.WBcel235.89” genome using STAR aligner version 2.6.0a. Read counts for each annotated gene were obtained using htseq-count (version 0.10.0) under the following settings: -f bam, -r pos, -s no, -m union, -t exon, and -i gene_id. Only uniquely mapped reads were used for downstream quantification. Normalization and differential expression analyses were performed using the Limma-Voom statistical framework, which models count data while accounting for heteroscedasticity. The Benjamini-Hochberg adjusted *P* values were used to assess statistical significance, with an adjusted *P* value <0.05 considered significant. The threshold of log_2_FC > 0.5 was used to identify genes up-regulated upon *cco-1* RNAi compared to empty vector (ev) at the same time point (e.g., *cco-1*_Day1 versus ev_Day1 and *cco-1*_Day11 versus ev_Day11). Functional annotation and gene enrichment analyses were conducted using DAVID (Database for Annotation, Visualization and Integrated Discovery) (*69*). Heatmaps of visualization of expression dynamics and clustering of DEGs in across time points were performed using the Morpheus platform (https://software.broadinstitute.org/morpheus).

### Bi-transcriptomic age clock analysis

To estimate transcriptomic age, normalized counts per million expression data were input into the publicly available BiT age prediction model (*43*), which uses an Elastic Net-selected set of 576 age-informative genes. The same model parameters and scripts as provided in the original BiT age publication were applied. Transcriptomic age predictions were generated across all six time points for both ev and *cco-1* RNAi–treated samples. These predicted values were subsequently used to assess aging trajectories and compare aging rates between treatments.

### Gene set enrichment analysis

Gene set enrichment was carried out using the GSEA algorithm implemented in the clusterProfiler R package, with gene sets derived from GO biological process and molecular function categories. Enrichment results were summarized by normalized enrichment scores (NESs) and adjusted *P* values (FDR). Significance was determined using an FDR < 0.05. To visualize dynamic pathway changes across time points, enriched pathways were plotted as dot plots showing NES values (color scale) and statistical significance [dot size, represented as –log_10_(FDR)].

### Binding motif enrichment analysis

Promoter sequences of selected genes spanning from −1000 bp upstream to +1000 bp downstream of the TSS were extracted, and de novo motif discovery was carried out using the findMotifs.pl script in HOMER (version 4.11) (*70*). Enriched motifs were ranked based on *P* value and the percentage of target genes containing each motif. The identified motifs were then annotated by comparison with known TF binding sites from the Cis-BP database (*71*), using the PWMEnrich R package (version 4.31.0). The genomic distributions of the enriched motifs were visualized using position-specific scoring matrices, and its positional bias relative to the TSS was evaluated. On the basis of the extracted promoter sequences, the genomic distribution of motif hits with a weight score > 6.0 was further analyzed using the pattern-matching function of the regulatory sequence analysis tools web server (http://rsat.eu) (*72*).

### ELT-2 sequence alignment and transcriptional activity reporter assay

For sequence alignment of ELT-2 (UniProt: Q10655) DNA binding sites, an amino acid sequence containing the zinc finger domain (amino acids 237 to 261) of ELT-2 was compared with human GATA1 (UniProt: P15976), GATA2 (UniProt: P23769), GATA3 (UniProt: P23771), GATA4 (UniProt: P43694), GATA5 (UniProt: Q9BWX5), and GATA6 (UniProt: Q92908) using Clustal Omega (*73*), followed by visualization of conserved residues with Jalview (version 2.11.2.7). The conserved arginine residues R249 and R250 were identified as candidate DNA binding sites. Site-directed mutagenesis was used to generate ELT-2 variants carrying point mutations (R249A and R250A) or a combined double mutant (R249/250A). Wild-type and mutant *elt-2* cDNAs were cloned into a c-Flag–tagged mammalian expression vector (pcDNA3.3). For transcriptional activity reporter assays, a pGL4.20-based GFP reporter plasmid was constructed in which four tandem repeats of the *cpr-5p-Motif_2* (ACTGATAAGA) were integrated into a minimal 70 bp promoter sequence: cccggACTGATAAGAcccaggACTGATAAGAcctgcggACTGATAAGAc-ctgcggACTGATAAGAcccca, to drive GFP expression. HEK293T cells were transiently cotransfected with ELT-2 expression plasmids (wild-type or mutants) together with the pGL4.20-based 4 x *cpr-5p-Motif_2::GFP* reporter using Lipo8000 transfection reagent (Beyotime, C0533). After 48 hours, GFP expression was examined by fluorescence microscopy and western blots.

### ELT-2 and DNA interaction modeling

To model the interaction between ELT-2 and the *cpr-5* promoter Motif_2 DNA, molecular docking was performed using the HADDOCK 2.4 web server (https://wenmr.science.uu.nl/haddock2.4/). The three-dimensional structure of the ELT-2 DNA binding domain (residues 151 to 286) was retrieved from UniProt entry Q10655 and modeled based on the template structure PDB ID 4hc9 using homology modeling. The DNA fragment corresponding to Motif-2 of the *cpr-5* promoter (sequence: 5′-ACTGATAAGA-3′) was generated in B-form conformation using the 3D-DART web server. Docking was performed with default HADDOCK parameters, using ambiguous interaction restraints derived from the known GATA-DNA binding interface. For each docking run, 200 initial structures were generated by rigid-body energy minimization, followed by semi-flexible simulated annealing in torsion angle space, and finally refinement in explicit water. The resulting complexes were clustered using a 7.5 Å root mean square deviation cutoff, and the best-scoring cluster was selected on the basis of the HADDOCK score. The wild-type ELT-2 model achieved a HADDOCK score of −236.54 (confidence score: 0.8495). The R249A mutant model was generated by in silico substitution of arginine 249 to alanine using PyMOL 3.0.1, followed by re-docking under identical conditions. All structural visualizations and interaction analyses were carried out using PyMOL 3.0.1 (Schrödinger, LLC). Putative hydrogen bonds and electrostatic interactions between the protein and DNA were identified with a distance cutoff of 4.0 Å.

### Cell culture and transient transfection

HEK293T (catalog no. CRL-3216, RRID: CVCL_0063) cells were obtained from American Type Culture Collection and maintained in Dulbecco’s modified Eagle’s medium containing 4.5 g of glucose per liter and 10% fetal bovine serum. All cell lines were validated to be free of mycoplasma contamination before use. Transient transfection of HEK293T cells were carried out with the Lipo8000 transfection reagent (Beyotime, C0533).

### Worm oxygen-consumption assays

Oxygen consumption was measured using the Seahorse XF96 equipment (Seahorse Bioscience) as described (*74*). Typically, 100 animals per condition were recovered from NGM plates with M9 medium, washed three times in 2 ml of M9 to eliminate residual bacteria, and resuspended in 500 μl of M9 medium. Worms were then transferred to 96-well standard Seahorse plates (catalog no. 100777-004, Seahorse Bioscience) (10 to 12 worms per well) and oxygen consumption was measured six times. Respiration rates were normalized to the number of worms in each individual well.

### qRT-PCR and ChIP-qPCR

Worms or cells were harvested, and total RNA was extracted using the same method described earlier for RNA-seq. For cDNA synthesis, 1000 ng of RNA was used with the Reverse Transcription Kit (catalog no. 205314, Qiagen). qRT-PCR was performed using the Light Cycler 480 SYBR Green I Master kit (catalog no. 04887352001, Roche). Primers for *pmp-3/act-3* and *Gapdh/Actin* were used as housekeeping for *C. elegans* and murine cells, respectively. ChIP-qPCR was performed as previously (*38*). In brief, OP56 [*elt-2::TY1::EGFP::3xflag*] worms were fixed in 1% formaldehyde solution for 15 min, followed by quenching with glycine. After 15 min of sonication, immunoprecipitations were carried out using anti-FLAG M2 beads (catalog no. A2220, Sigma-Aldrich) in RIPA buffer. Primers used for qRT-PCR and ChIP-qPCR are listed in table S4.

### Compounds treatment in *C. elegans*

In addition to NGM, a final concentration of 1 or 4 mM CQ (catalog no. C6628, Sigma-Aldrich), tunicamycin (5 μg/ml; A611129, Sangon), and 0.1 mM auxin IAA (catalog no. A10556, Alfa Aesar) was added just before pouring the plates. For compound stocks, CQ was dissolved in M9 buffer [Na_2_HPO_4_ (6 g/liter), KH_2_PO_4_ (3 g/liter), NaCl (5 g/liter), and 1 M MgSO_4_ (1 ml/liter) in distilled water] (400 mM), tunicamycin (5 mg/mL) was dissolved in dimethyl sulfoxide, and IAA (100 mM) was dissolved in ethanol kept at −80°C.

### Worm imaging and quantification of fluorescent signals

To ensure consistent imaging conditions and enable quantitative analysis of fluorescent signals, a standardized worm alignment-based imaging protocol was employed. In brief, 8 to 10 *C. elegans* were randomly selected from each treatment group and transferred into a 10-μl drop of 10 mM tetramisole (catalog no. A506410, Sangon) on a 2% agarose pad for immobilization. Worms were carefully aligned in a lateral orientation using a fine eyelash or platinum wire picker to minimize variability in image acquisition. Fluorescence images were captured using a Nikon SMZ1000 fluorescence stereomicroscope under identical exposure settings across all experimental conditions. The fluorescent number and intensity of aggregates were quantified with Fiji/ImageJ software (version 1.53c) by defining a consistent region of interest and measuring integrated density values.

### Lifespan analysis

Lifespan assays were performed as previously described (*38*). In brief, synchronized young adult hermaphrodites were transferred to NGM plates seeded with *E. coli* HT115 expressing either an empty vector (ev) or specific RNAi constructs. Following a 48-hour egg-laying period, adult worms were removed to generate age-matched populations. The progenies were maintained at 20°C until the young adult stage. Subsequently, 80 to 100 L4-stage worms per condition were transferred to fresh RNAi plates. Lifespan scoring was conducted every other day. Worms were classified as dead when they failed to respond to three times of gentle poking with a worm picker. Animals that exhibited vulval rupture, internal hatching, or desiccation from crawling off the plate were censored from the analysis. To ensure reproducibility, all lifespans were examined in at least three biological replicates with 80 to 100 worms in each replicate. Paralysis analyses were manually scored by poking, at least 80 total worms were analyzed for each condition. Statistical analyses and details of replication for all lifespan experiments conducted in the current study are provided in table S3.

### LysoSensor and LysoTracker staining

To assess lysosomal acidity, approximately 40 worms per condition were collected on days 1, 5, and 8 of adulthood after being maintained on RNAi or control plates. Worms were washed with M9 buffer and incubated in 80 μl of S-basal buffer supplemented with 10 μM LysoSensor Green DND-189 (catalog no. L7535, Thermo Fisher Scientific) and/or 10 μM LysoTracker Red DND-99 (catalog no. L7528, Thermo Fisher Scientific). Staining was performed at 20°C in the dark for 1 hour. Following incubation, worms were transferred to seeded NGM plates for 1 hour of recovery under dark conditions. Animals were then mounted and imaged using a ZEISS LSM 880 confocal microscope equipped with Airyscan 2 for high-resolution acquisition. Quantification of fluorescence intensity was conducted using Fiji software (version 1.53c), and the relative signal was shown as LysoSensor Green versus LysoTracker Red ratios.

### Protein extraction for Western blots and filter trap assay

Protein extracts were prepared using RIPA buffer supplemented with protease and phosphatase inhibitors. Protein concentrations were then measured and normalized on the basis of the bicinchoninic acid assay. Equal amounts of protein were separated via SDS–polyacrylamide gel electrophoresis and transferred onto polyvinylidene difluoride membranes. Membranes were blocked with 5% bovine serum albumin (BSA) and incubated with the following primary antibodies: anti-GFP (catalog no. 2956, CST, 1:1000, RRID: AB_1196615), anti-Flag (catalog no. F1804, Sigma-Aldrich, 1:2000, RRID: AB_262044), anti-tubulin (catalog no. T5168, Sigma-Aldrich, 1:2,000, RRID: AB_477579), anti-actin (Proteintech, 66009-1-Ig, 1:10,000, RRID: AB_2687938), and anti–CPL-1 (1:5000; gift from X. Wang). The horseradish peroxidase–labeled anti-rabbit (catalog no. 7074; CST, 1:5000, RRID: AB_2099233) and anti-mouse (catalog no. 5656T; CST; 1:5,000, RRID: AB_330924) secondary antibodies were applied afterward.

For filter trap assay, following protein extraction with a non-denaturing lysis buffer, the protein solution was mixed 1:1 (v/v) with 1% SDS and incubated at 25°C for 10 min (*75*). Protein samples (100 μg) were loaded on to nitrocellulose membrane with 0.45-μm pore size (Sigma-Aldrich, catalog no. HATF00010), assembled in vacuum slot blotter (Bio-Dot, Bio-Rad). The membrane was then washed three times with 200 μl of 0.2% SDS and sealed with 5% BSA. Immunoblotting was performed using anti-GFP (catalog no. 2956, CST, 1:1,000, RRID: AB_1196615) antibody following classical Western blotting procedures.

### Study design and statistical analysis

Sample sizes were determined on the basis of prior publications and expected effect sizes. All experiments (excluding RNA-seq) were independently repeated at least twice and similar results were acquired. Except for the random allocation of *C. elegans* to experimental groups/treatments after large-scale synchronization, the experiments were not randomized. No animals were excluded except for predefined criteria in lifespan assays (e.g., ruptured vulva and desiccation). Investigators were not blinded during experiments or data analysis. Quantitative data were analyzed using GraphPad Prism 8 (version 8.3.1) or JMP 18 (version 18.0.1). For two-group comparisons, unpaired two-tailed Student’s *t* tests were used. One-way or two-way analysis of variance (ANOVA) followed by Tukey’s post hoc test was applied for comparisons involving multiple groups or variables. Kaplan-Meier survival curves were compared using the log-rank (Mantel-Cox) test. Data are presented as mean ± SEM unless otherwise specified.
