# Supplementary material for: Mitochondrial stress activates ELT-2–dependent lysosomal proteostasis to extend lifespan in C. elegans
Source: Sci Adv. 2026 Aug 12;12(33):eaeh0657. doi: 10.1126/sciadv.aeh0657 (PMC13464485; doi:10.1126/sciadv.aeh0657)
Supplement: Supplementary file 1 [file sciadv.aeh0657_sm.pdf]

Science Advances
AAAS

Supplementary Materials for

Mitochondrial stress activates ELT-2–dependent lysosomal proteostasis to extend lifespan in *C. elegans*

Rendan Yang *et al.*

Corresponding author: Liangshan Mu, mu.liangshan@zs-hospital.sh.cn; Johan Auwerx, admin.auwerx@epfl.ch; Terytty Yang Li, teryttyliyang@fudan.edu.cn

Sci. Adv. **12**, eaeh0657 (2026)
DOI: 10.1126/sciadv.aeh0657

The PDF file includes:

Figs. S1 to S10
Legends for tables S1 to S4

Other Supplementary Material for this manuscript includes the following:

Tables S1 to S4

Supplementary Figures

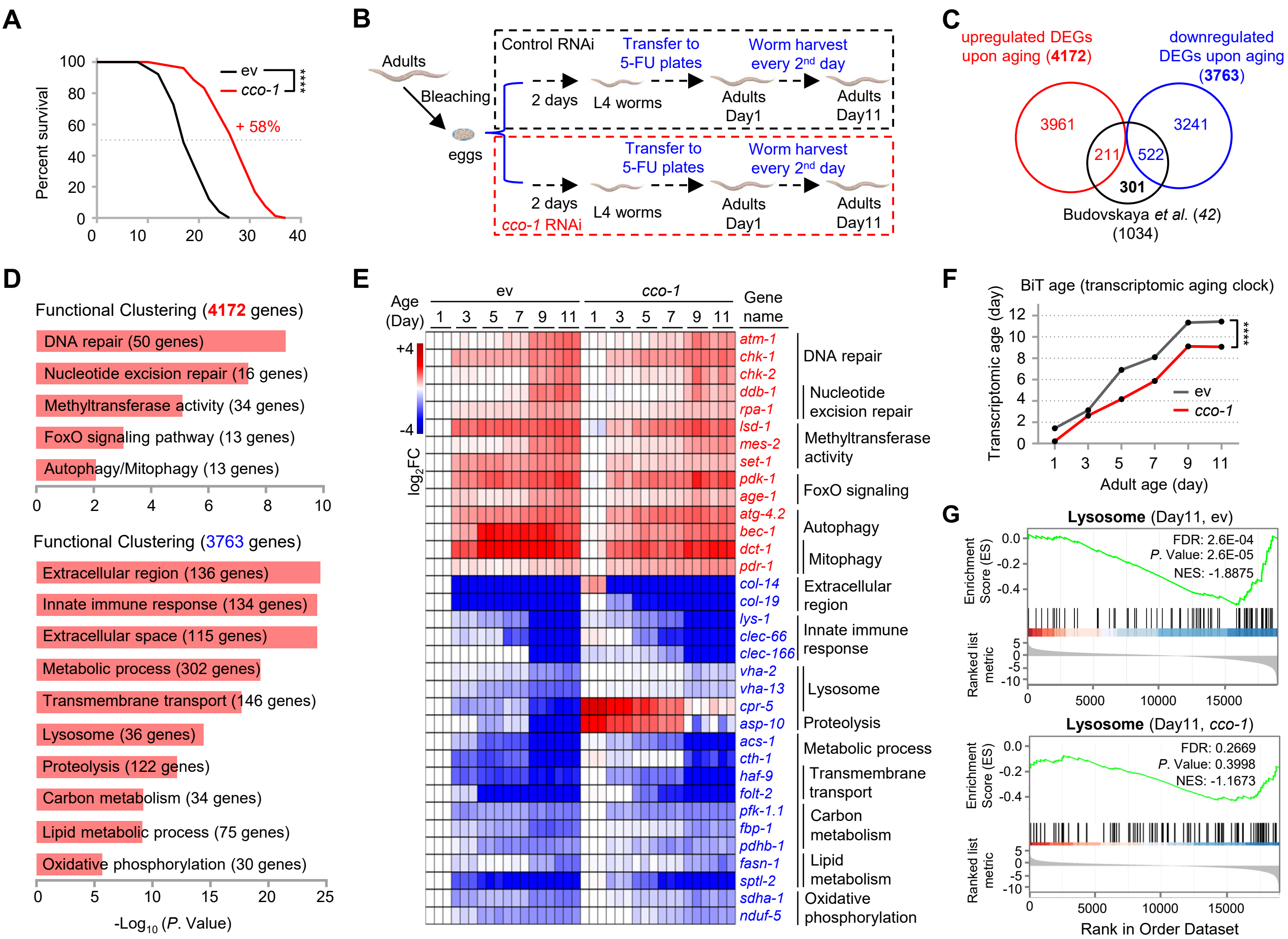

Fig. S1. Time-dependent tracking of aging-associated transcriptional signatures with or without mitochondrial respiration inhibition in *C. elegans*. (**A**) Survival of worms fed with control (ev) or *cco-1* RNAi. (**B**) Schematic diagram showing the experimental design for time-dependent tracking of the aging-associated transcriptional signatures in *C. elegans* fed with control (ev) or *cco-1* RNAi. (**C**) Venn diagram of the up- and downregulated differentially expressed genes (DEGs) in aged (Day 9 and Day 11) worms, as compared to young (Day 1) worms fed with control RNAi, and a previous aging transcriptome study (*42*). (**D**) Functional clustering of the 4,172 and 3,763 DEGs as indicated in (C). (**E**) Some representative genes that differentially expressed upon aging (log_2FC relatively to control RNAi at Day 1). The up- and downregulated DEGs upon aging are highlighted in red and blue, respectively. (**F**) BiT aging Clock analysis (n = 3) of the transcriptome data validate that *cco-1* RNAi worms were biologically younger. (**G**) Gene set enrichment analysis (GSEA) of Lysosome genes in aged (Day 11) worms as compared to that in young (Day 1) worms fed with control or *cco-1* RNAi. FDR, false-discovery rate. NES, normalized enrichment score. Statistical analysis was performed by log-rank test (A) or ANOVA followed by Tukey post-hoc test (F) (****P < 0.0001). Statistical data for lifespan can be found in table S3.

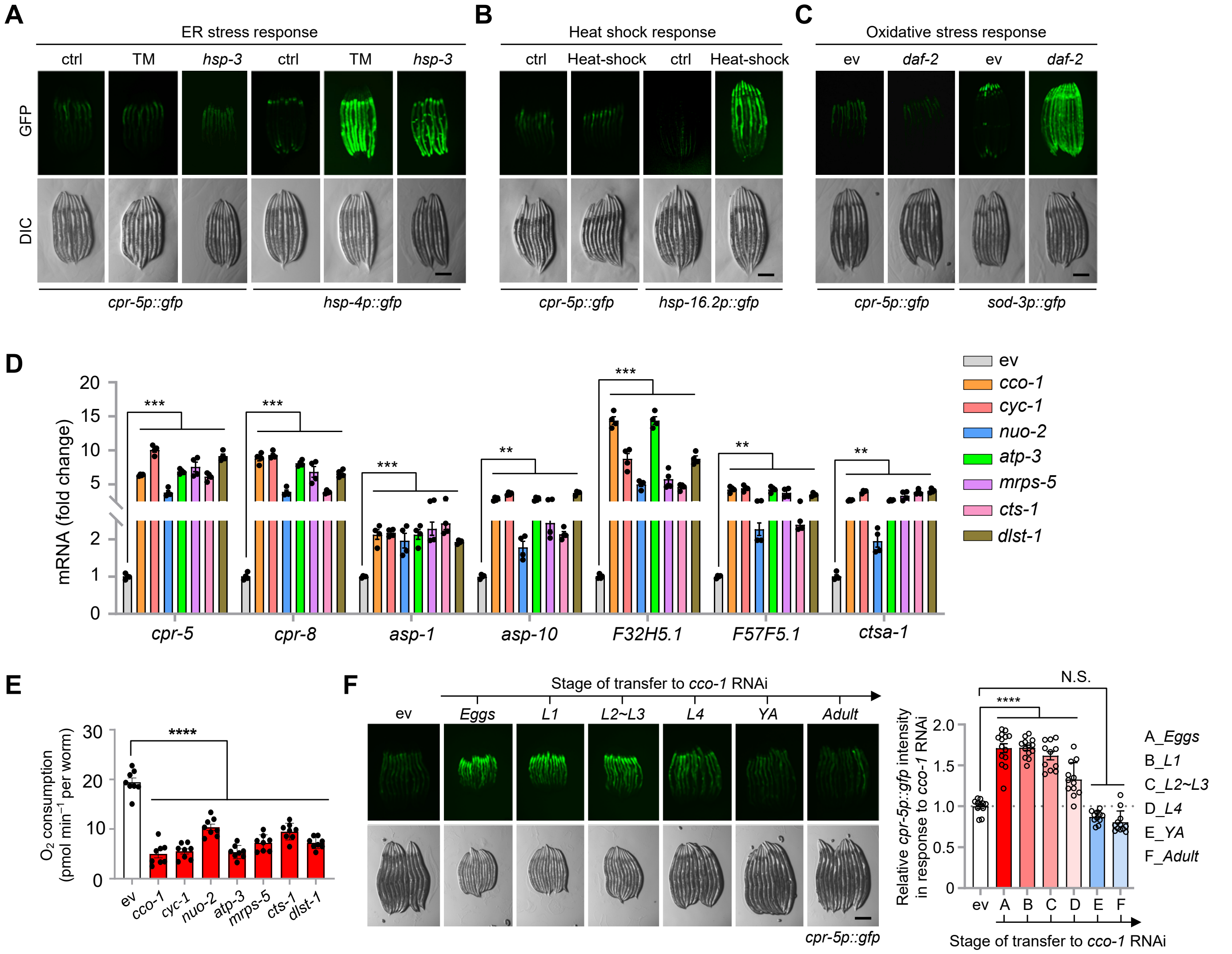

Fig. S2. Mitochondrial stress but not other classical stress response inducers induced the expression of lysosomal genes in *C. elegans*. (**A**) ER stress response inducers tunicamycin (5 µg/ml) and *hsp-3* RNAi induced the GFP expression in *hsp-4p::gfp*, but not in *cpr-5p::gfp*, worms. (**B**) Heat shock (30°C for 6 h) induced the GFP expression in *hsp-16.2p::gfp*, but not in *cpr-5p::gfp*, worms. (**C**) Oxidative stress response inducer *daf-2* RNAi induced the GFP expression in *sod-3p::gfp,* but not in *cpr-5p::gfp*, worms. (**D**) qRT-PCR analysis (n = 4 biologically independent samples) of transcripts in worms fed with control (ev), *cco-1, cyc-1, nuo-2, atp-3*, *mrps-5, cts-1* and *dlst-1* RNAi. (**E**) The basal respiration of adult day 1 worms fed with RNAi as indicated in (D) (n = 8 biologically independent samples). (**F**) *C. elegans* expressing *cpr-5p::gfp* were shifted from control (ev) to *cco-1* RNAi at different developmental stages (Eggs, L1, L2-L3, L4, YA, Adult). Relative GFP intensity was then quantified (n = 11-15 independent worms for each condition). Error bars denote SEM. Scale bars, 0.3 mm. Statistical analysis was performed by ANOVA followed by Tukey post-hoc test (**$P < 0.01$; ***$P < 0.001$; ****$P < 0.0001$; N.S., not significant).

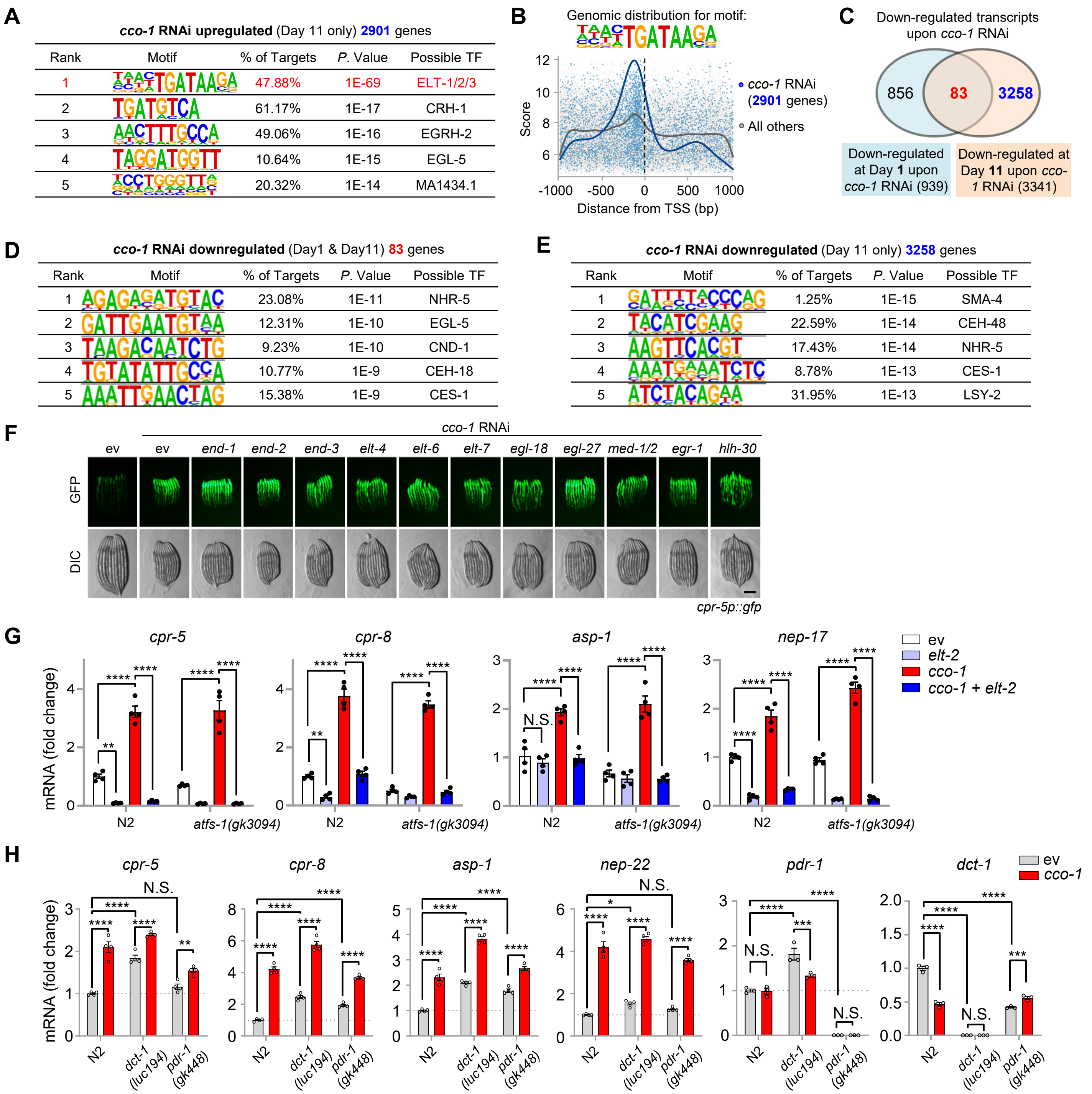

Fig. S3. Identification of ELT-2 as a key regulator for the transcriptional response in response to mitochondrial respiration inhibition. (**A**) The top five enriched motifs for promoters of the 2,901 upregulated genes upon *cco-1* RNAi at Day 11, by using Hypergeometric optimization of motif enrichment (HOMER) and ranked based on the *P* values. *P*. Value was derived from HOMER (one-sided hypergeometric test). (**B**) The genomic distribution of the motif hits at the promoters of 2,901 upregulated genes upon *cco-1* RNAi, and all other genes. Scores are assigned based on the position weight matrix (PWM) of the motif. Lines indicate the density of the motif hits. TSS, transcription start site. (**C**) Venn diagram of the downregulated transcripts in response to *cco-1* RNAi treatment in young (Day 1) and aged (Day 11) worms. (**D** and **E**) The top five enriched motifs for promoters of the downregulated genes upon *cco-1* RNAi at Day 1 & Day 11 (D) or at Day 11 only (E), by using HOMER and ranked based on the *P* values (one-sided hypergeometric test). (**F**) RNAi of other GATA transcription factor members, or *hlh-30*, did not affect the GFP expression of *cpr-5p::gfp* worms induced by *cco-1* RNAi. RNAi targeting *cco-1* occupied 40%, RNAi targeting GATA transcription factor members, or *hlh-30*, occupied 60%. Scale bar, 0.3 mm. (**G**) qRT-PCR analysis (n = 4 biologically independent samples) of wild-type (N2) and *atfs-1(gk3094)* worms fed with control, *cco-1* and/or *elt-2* RNAi. (**H**) qRT-PCR analysis (n = 4 biologically independent samples) of wild-type (N2), *dct-1(luc194)* and *pdr-1(gk448)* worms fed with control or *cco-1* RNAi. Error bars denote SEM. Statistical analysis was performed by ANOVA followed by Tukey post-hoc test (*P < 0.05; **P < 0.01; ***P < 0.001; ****P < 0.0001; N.S., not significant).

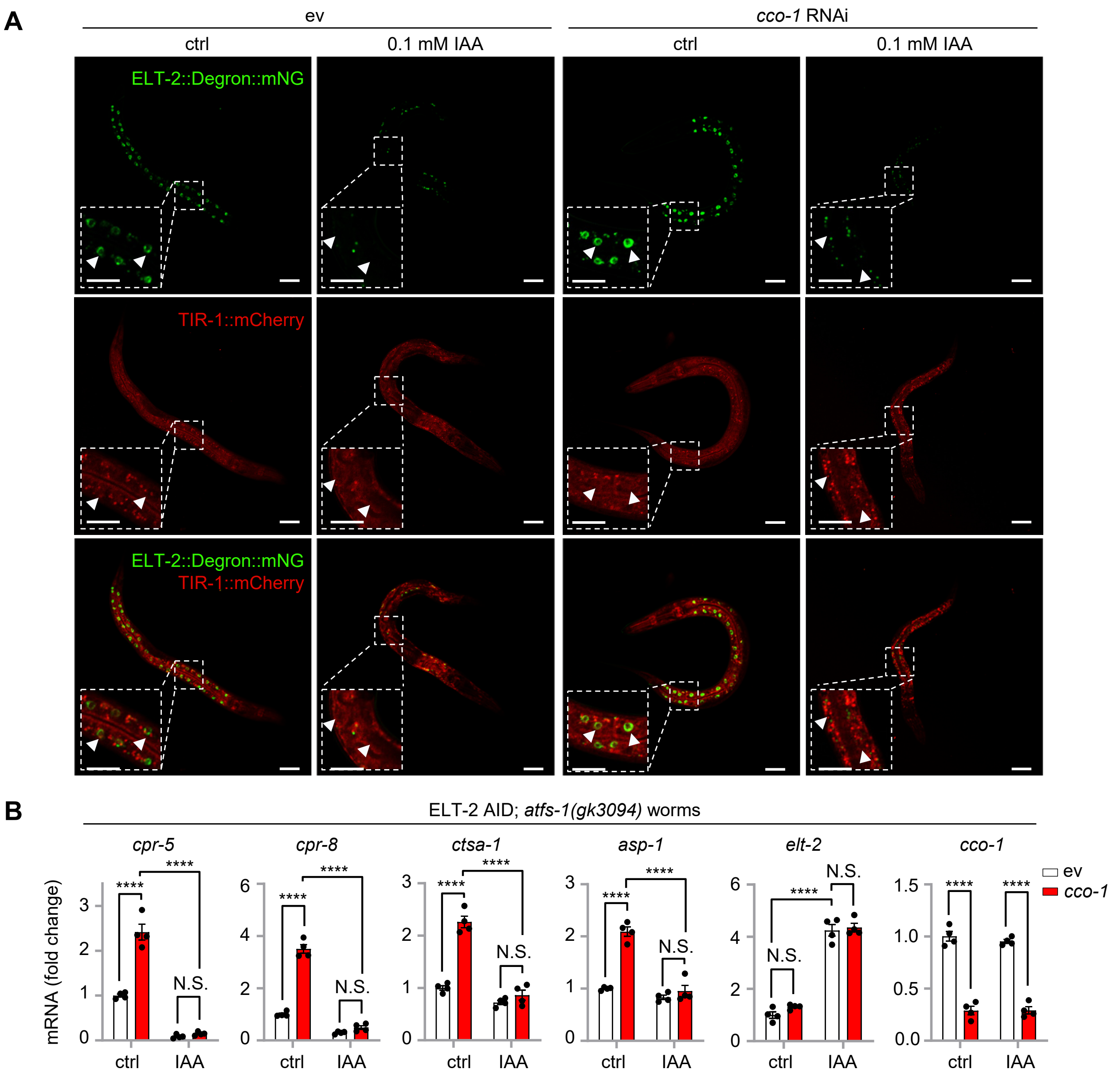

Fig. S4. Auxin-inducible degradation (AID) of ELT-2 mimics the effect of *elt-2* RNAi in suppressing ELT-2 dependent-MSR activation upon mitochondrial stress. (**A**) The natural auxin indole-3-acetic acid (IAA) treatment at 0.1 mM remarkably decreased the Degron-mNG-ELT-2 expression in the ELT-2 AID (*elt-2::Degron::mNeonGreen;eft-3p::TIR1::mRuby*); *atfs-1(gk3094)* worms. White arrows indicate the nucleus where the Degron-mNG-ELT-2 signals typically accumulate. Scale bars, 0.1 mm. (**B**) qRT-PCR analysis (n = 4 biologically independent samples) of ELT-2 AID; *atfs-1(gk3094)* worms treated with control (ctrl) or *cco-1* RNAi and/or 0.1 mM IAA. Error bars denote SEM. Statistical analysis was performed by ANOVA followed by Tukey post-hoc test (****P < 0.0001; N.S., not significant).

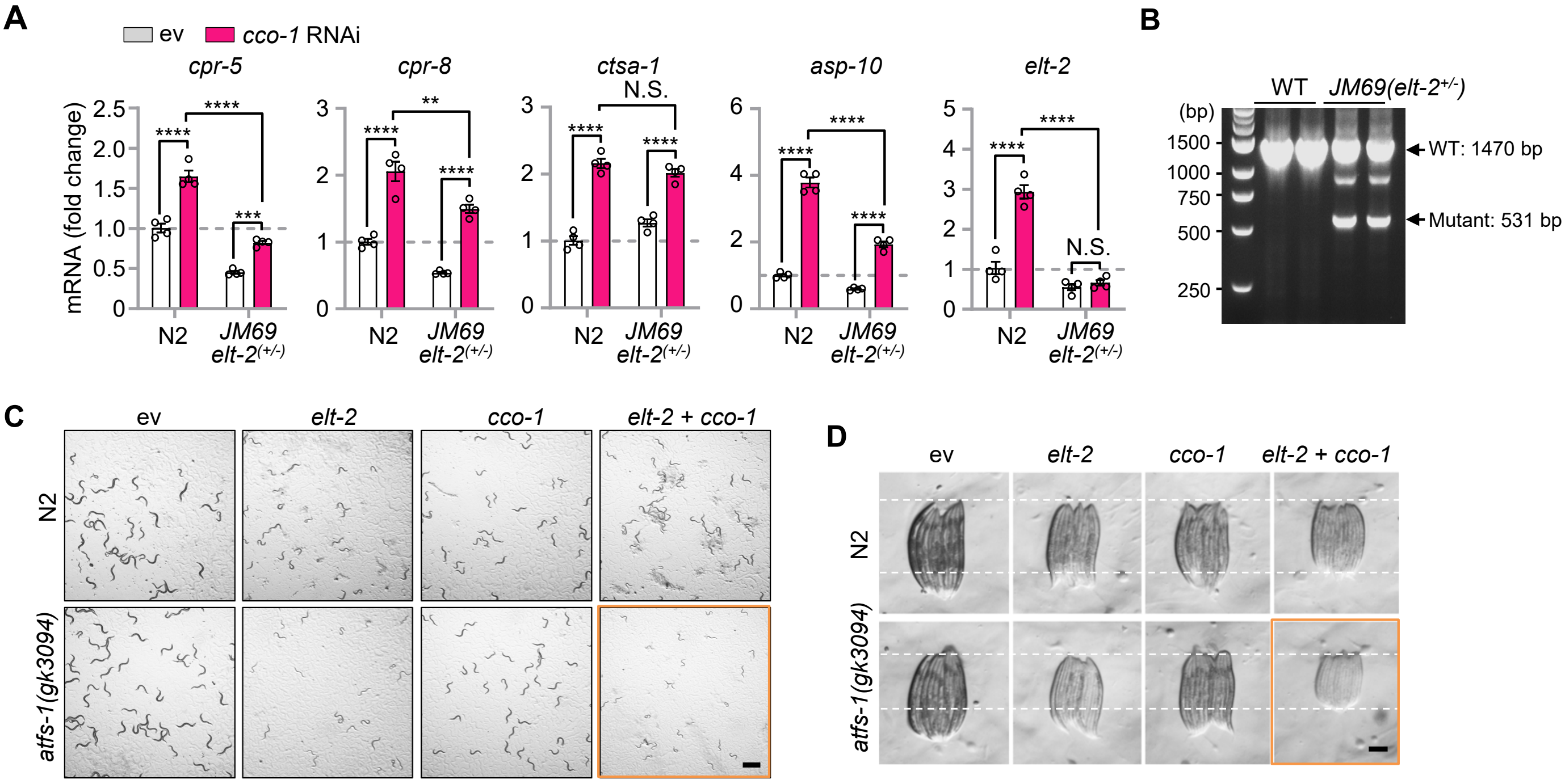

Fig. S5. Impacts of *elt-2* mutation or RNAi on mitochondrial stress-induced lysosomal gene expression and worm body size. (**A**) qRT-PCR analysis (*n* = 4 biologically independent samples) of wild-type (N2) and *JM69(elt-2$^{+/-}$)* worms fed with empty vector (ev) or *cco-1* RNAi. (**B**) Genotyping results for the wild-type and *elt-2* heterozygous *JM69(elt-2$^{+/-}$)* worm strains. (**C**) Representative photos of wild-type (N2) and *atfs-1(gk3094)* worms fed with control, *cco-1* and/or *elt-2* RNAi since the maternal L4 stage. Scale bar, 1 mm. (**D**) Alignment results of worms as indicated in (C). Scale bar, 0.3 mm. Error bars denote SEM. Statistical analysis was performed by ANOVA followed by Tukey post-hoc test (**$P < 0.01$; *** $P < 0.001$; ****$P < 0.0001$; N.S., not significant).

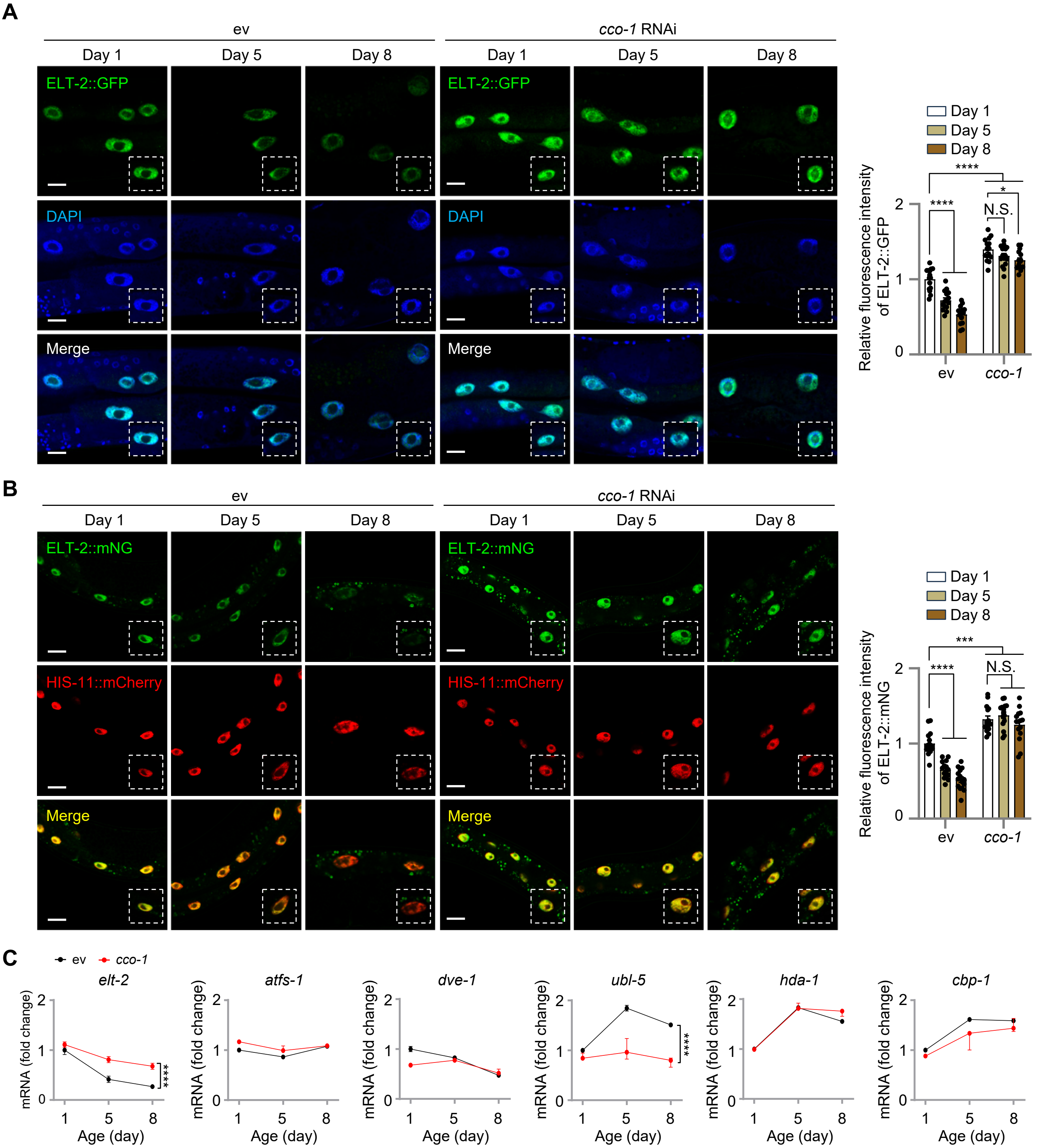

Fig. S6. Aging-associated down-expression of ELT-2 is suppressed in worms with mitochondrial respiration inhibition. (**A**) Representative images of exogenous GFP-tagged ELT-2 and DAPI staining in *elt-2p::elt-2::gfp-flag* worms fed with control or *cco-1* RNAi, and analyzed at different adult ages. Scale bars, 10 µm. The fluorescence intensity of ELT-2::GFP was quantified (n = 15 nuclei for each condition). (**B**) Representative images of endogenous mNG-tagged ELT-2 and mCherry-tagged histone in *elt-2::Degron::mNeonGreen; HIS-11::mCherry* worms fed with control or *cco-1* RNAi, and analyzed at different adult ages. Scale bars, 10 µm. The fluorescence intensity of ELT-2::mNG was quantified (n = 15 nuclei for each condition). (**C**) The mRNA levels of indicated genes (n = 4 biologically independent samples) of worms fed with control or *cco-1* RNAi, collected at different ages. Error bars denote SEM. Statistical analysis was performed by ANOVA followed by Tukey post-hoc test (*$P < 0.05$; ***$P < 0.001$; ****$P < 0.0001$; N.S., not significant).

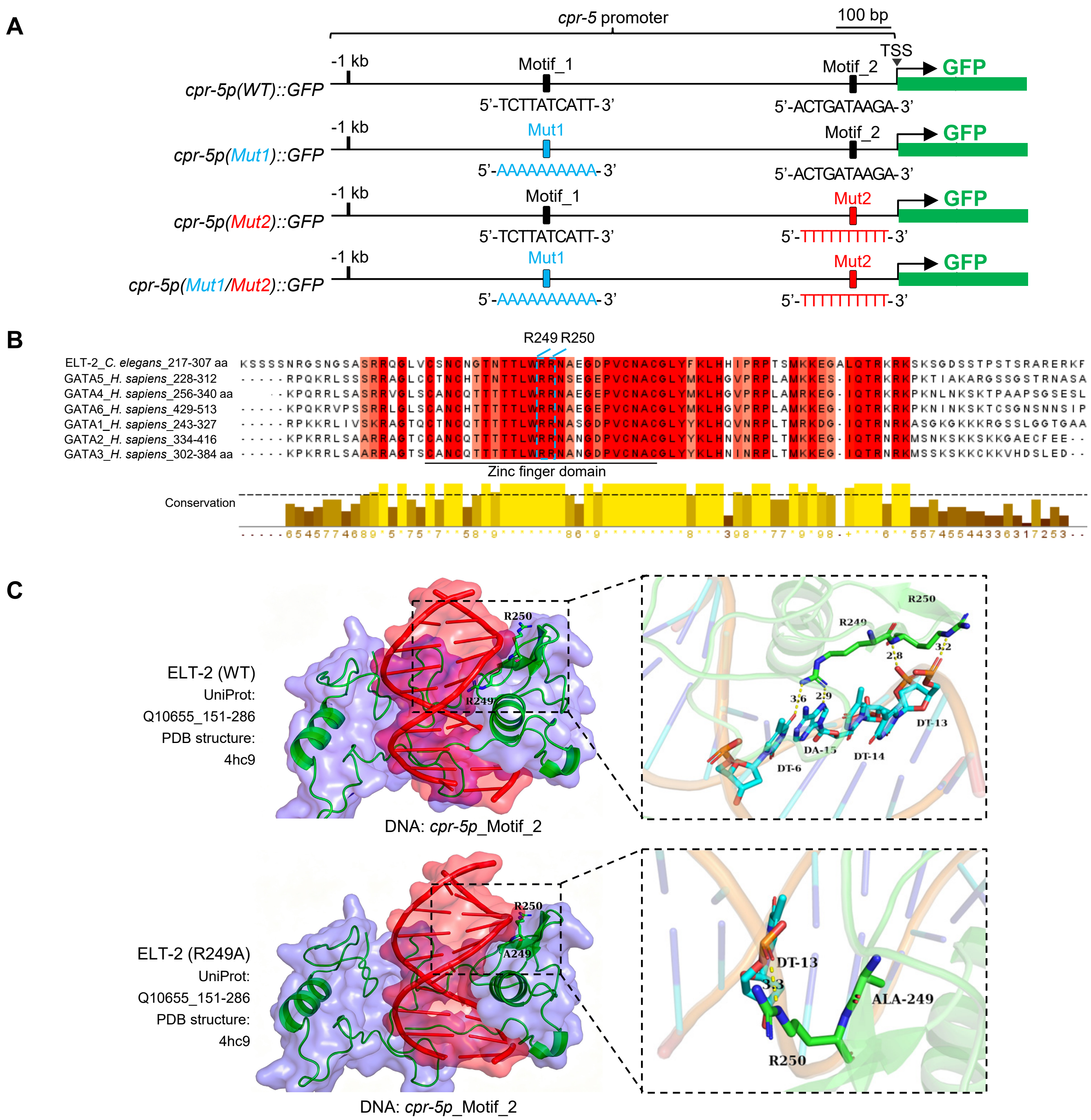

Fig. S7. The putative ELT-2 binding motifs in *cpr-5* promoter and the key DNA binding sites of ELT-2. (**A**) Schematic diagram of the generation of four transgenic worms carrying the wild-type (WT) or mutated *cpr-5* promoter driving GFP reporter constructs. Two putative ELT-2 binding motifs (Motif_1 and Motif_2) were identified within 1 kb upstream of the transcription start site (TSS) of *cpr-5* gene. These two motifs were individually or combinedly mutated to A/T to generate *cpr-5p(Mut1)::GFP*, *cpr-5p(Mut2)::GFP* and *cpr-5p(Mut1/Mut2)::GFP* reporter constructs. (**B**) Sequence alignment of ELT-2 with human GATA family members revealed conserved arginine residues (R249 and R250) within the zinc finger domain. (**C**) Structural features of the interactions between wild-type or R249A mutant forms of ELT-2 (UniProt: Q10655) and the *cpr-5* promoter Motif-2 (DNA sequence 5'-ACTGATAAGA-3'). ELT-2 was shown as cartoon/surface in purple/green, and DNA in red. Key residues were highlighted as sticks. The following interactions were found: for WT-ELT-2, R249 forms putative hydrogen bonds/electrostatic interactions with DT-6 and DA-15 of the DNA, with distances of 3.6 Å and 2.9 Å, respectively; R250 interacts with DT-14 and DT-13 of the DNA, with distances of 2.8 Å and 3.2 Å, respectively. For R249A-ELT-2, only the putative hydrogen bond/electrostatic interaction between R250 and DT-13 of the DNA was retained, with a distance of 3.3 Å. Yellow dashed lines indicate these interactions, with distances given in ångströms (Å).

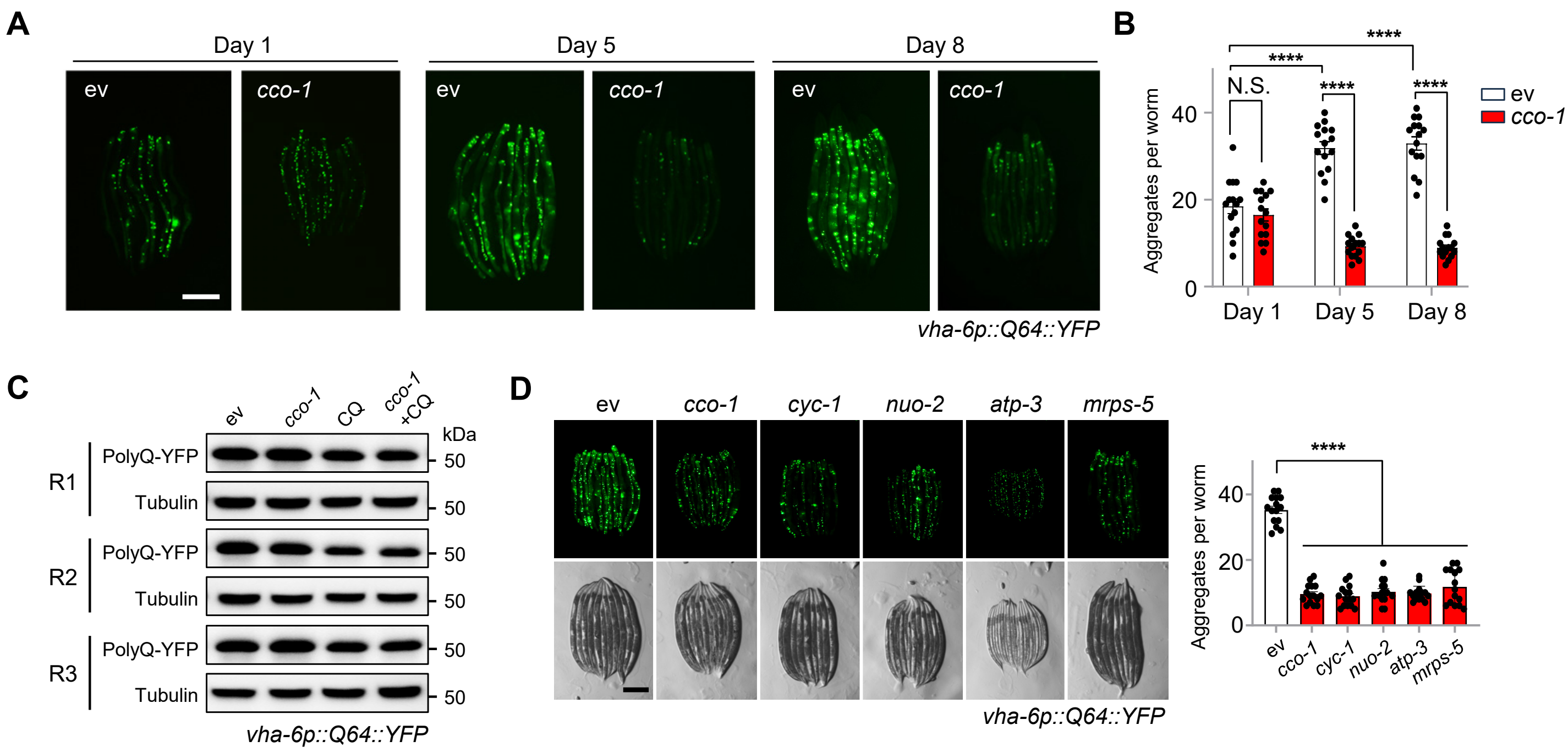

Fig. S8. Mitochondrial stress reduces aging-associated intestinal polyQ aggregate accumulation. (**A**) RNAi of *cco-1* reduces the aggregation formation in *vha-6p::Q64::YFP* worms. Worms expressing *vha-6p::Q64::YFP* were treated with control or *cco-1* RNAi, and analyzed at different adult ages. Scale bar, 0.3 mm. (**B**) Quantification of the number of polyQ aggregates in worms as indicated in (A) (n = 15 individual worms for each condition). (**C**) Western blots of the total cell lysates of *vha-6p::Q64::YFP* worms fed with control or *cco-1* RNAi and/or 1 mM CQ at adult day 5. R1-R3 indicates three independent replicates. (**D**) Worms expressing *vha-6p::Q64::YFP* were treated with indicated RNAi, and analyzed at adult day 5. Scale bar, 0.3 mm. The number of aggregates per worm was quantified (n = 15 individual worms for each condition). Error bars denote SEM. Statistical analysis was performed by ANOVA followed by Tukey post-hoc test (****$P < 0.0001$; N.S., not significant).

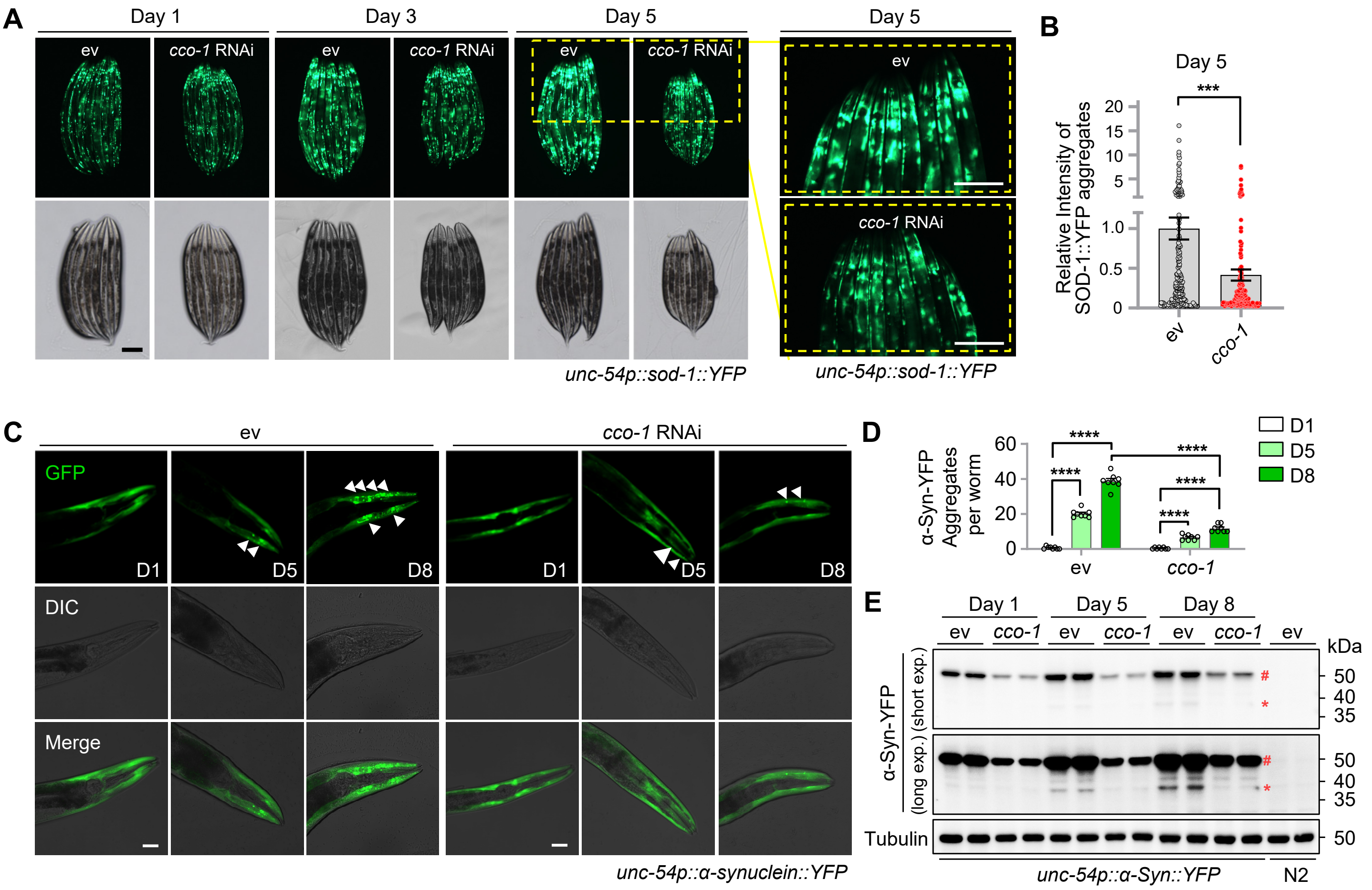

Fig. S9. Mitochondrial stress suppresses muscular SOD-1 and α-synuclein protein aggregation formation upon aging. (**A**) Fluorescence microscopy images of AM725[*unc-54p::sod-1::YFP*] worms fed with empty vector control (ev) or *cco-1* RNAi, examined on Adult Day 1, 3 and 5. Regions outlined by the yellow box were shown at higher magnification on the right. Scale bars, 0.3 mm. (**B**) Quantification of relative SOD-1::YFP fluorescence intensity (n = 236 aggregates for ev; 188 aggregates for *cco-1* RNAi) of Adult Day 5 worms as indicated in (A). (**C**) Representative confocal fluorescence images of NL5901[*unc-54p::α-synuclein::YFP*] worms fed with control or *cco-1* RNAi, examined on Day 1, 5 and 8. Arrows indicate typical pharyngeal fluorescent aggregates. Scale bars, 0.1 mm. (**D**) The number of α-Synuclein aggregates (n = 8 individual worms for each condition) of worms as indicated in (C) was quantified. (**E**) Western blots of α-Synuclein::YFP levels in worm lysates treated as in (C). Crosshatch (#) and asterisk (*) indicate the monomeric and sub-monomeric forms of α-Synuclein, respectively. Wild-type (N2) worms serve as background control. Error bars denote SEM. Statistical analysis was performed by ANOVA followed by two-tailed and unpaired Student's t-test (B) or Tukey post-hoc test (D) (***$P < 0.001$; ****$P < 0.0001$; N.S., not significant).

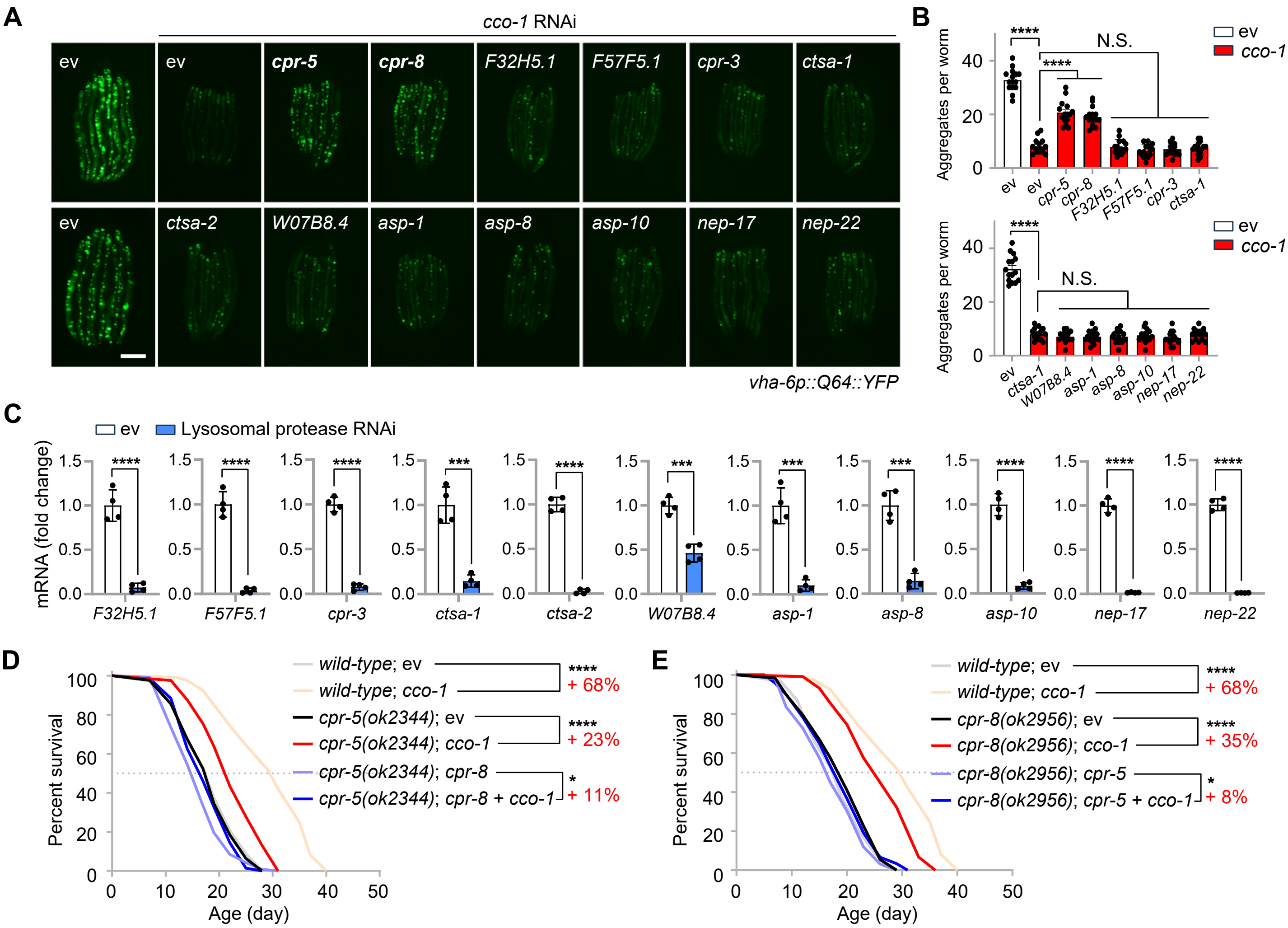

Fig. S10. Impacts of *cco-1* RNAi and lysosomal proteases silencing in polyQ aggregation clearance and lifespan extension. (**A**) Worms expressing *vha-6p::Q64::YFP* were treated with control or *cco-1* (50%) RNAi, combined with RNAi targeting the lysosomal protease genes (50%). Scale bar, 0.3 mm. (**B**) Quantification of the number of polyQ aggregates in worms as indicated in (E) (*n* = 15 individual worms for each condition). (**C**) qRT-PCR analysis (*n* = 4 biologically independent samples) of worms fed with control or RNAi targeting the lysosomal protease genes (50%). (**D**) Survival of wild-type (N2) and *cpr-5(ok2344)* worms fed with control, *cco-1* (50%) and/or *cpr-8* (50%) RNAi. The impacts of *cco-1* RNAi on the mean lifespan extension in different backgrounds were as indicated in red numbers. (**E**) Survival of wild-type and *cpr-8(ok2956)* worms fed with control, *cco-1* (50%) and/or *cpr-5* (50%) RNAi. Error bars denote SEM. Statistical analysis was performed by ANOVA followed by Tukey post-hoc test (B), by two-tailed and unpaired Student's *t*-test, or log-rank test (D and E) (**P* < 0.05; ****P* < 0.001; *****P* < 0.0001; N.S., not significant). Statistical data for lifespan can be found in table S3.

Table S1. RNA-seq results of worms fed with control or *cco-1* RNAi and harvested at different ages.

Table S2. RNA-seq results of wild-type and *atfs-1(gk3094)* worms fed with *cco-1* and/or *elt-2* RNAi.

Table S3. Summary of lifespan experiments in this study.

Table S4. List of primers used for qRT-PCR, ChIP-qPCR and genotyping in this study.
